# Luminescent Guests Encapsulated in Metal–Organic Frameworks for Portable Fluorescence Sensor and Visual Detection Applications: A Review

**DOI:** 10.3390/bios13040435

**Published:** 2023-03-29

**Authors:** Xu Xu, Muyao Ma, Tongxin Sun, Xin Zhao, Lei Zhang

**Affiliations:** 1 College of Chemistry, Liaoning University, No. 66 Chongshan Middle Road, Shenyang 110036, China; 2 Ecology and Environmental Monitoring Center of Jilin Province, Changchun 130011, China

**Keywords:** metal–organic frameworks, fluorescence, luminescent guests, portable visual application

## Abstract

Metal–organic frameworks (MOFs) have excellent applicability in several fields and have significant structural advantages, due to their open pore structure, high porosity, large specific surface area, and easily modifiable and functionalized porous surface. In addition, a variety of luminescent guest (LG) species can be encapsulated in the pores of MOFs, giving MOFs a broader luminescent capability. The applications of a variety of LG@MOF sensors, constructed by doping MOFs with LGs such as lanthanide ions, carbon quantum dots, luminescent complexes, organic dyes, and metal nanoclusters, for fluorescence detection of various target analyses such as ions, biomarkers, pesticides, and preservatives are systematically introduced in this review. The development of these sensors for portable visual fluorescence sensing applications is then covered. Finally, the challenges that these sectors currently face, as well as the potential for future growth, are briefly discussed.

## 1. Introduction

Accompanied by the development of society and industrial production, food safety, environmental monitoring, and clinical diagnostics, the most important global issues, have attracted extensive attention worldwide. In many applications, it is increasingly important to accurately detect and measure biological and chemical substances [1,2,3,4,5]. Conventional laboratory techniques are typically unsuitable for rapid on-site analysis, because they require heavy equipment and skilled operators. In particular, the cost of equipment will severely limit the use of these conventional approaches in less developed countries and regions. It is therefore essential to develop a lightweight, affordable device for on-site detection.

In recent years, various fast detection techniques have been established for sensitive detection in food safety, environment surveillance, and clinical diagnosis, such as electrochemical analytical methods [6,7,8,9,10], fluorescent analytical methods [11,12,13], surface-enhanced Raman spectroscopy [14,15,16,17,18], and so forth [19]. Due to their high sensitivity, ease of use, and rapid response, fluorescent sensing techniques have been widely used for the detection of metal ions [20,21,22,23,24,25], pesticide residues [26,27,28,29,30,31,32,33], disease markers [34,35,36,37,38], etc. In particular, they have great potential for integrating fluorescent probes and associated detection accessories into portable monitoring systems, such as systems that determine the visual readability of analytes, by using a simple ultraviolet (UV) lamp [39]. As a result, fluorescence-based analysis is often a better choice, and developing materials with good fluorescence signals and active sites of detection is more suitable for integration into fluorescence sensors.

Metal–organic framework (MOF) materials, with the advantages of porosity, large specific surface area, structural and functional diversity, and unsaturated metal sites, have attracted much attention in the sensing field, as the standout material for fluorescence detection [40,41,42]. The luminescence of MOFs can originate from the ligands, metal ions, or the interaction between ligands and metal ions. Many types of sensors are used, such as ZIF-8, ZIF-67, Ln-MOFs, and UiO-66 [43]. Although MOFs have been widely used for sensing, lanthanide metal ions and complex organic linkers are the main sources of their tunable fluorescence properties. This suggests that their synthesis will be extremely challenging [44,45,46,47]. Thus, imparting better fluorescence properties remains a great challenge.

In addition to the luminescence of the MOF itself, the luminescence of MOFs can be realized by introducing luminous guests into the metal–organic framework. Due to their structural properties, MOFs are an excellent class of hosts, and by adding guests, their variety of functions and practical applications can be modified. Using the porosity of MOF hosts to encapsulate various luminescent guests (LGs), thus forming LG@MOF composite systems, is a unique approach to obtaining luminescence from MOFs (LMOFs). Over conventional syntheses of luminescent materials, guest encapsulation into MOFs has some benefits, including the simplicity and cost-effectiveness of this methodology, the possibility of tuning the emission properties by a rational selection of commercially available fluorophores or luminescent dyes, and the avoidance of aggregation-caused quenching (ACQ) phenomena, by partitioning of the luminescent guests into the pores of the crystalline MOF host [48,49,50,51,52,53,54]. LG@MOFs not only combine the advantages of LGs and MOFs but also maintain the original morphological characteristics. The adsorption ability of MOFs makes the analyte concentrate near the fluorescence sensor, to improve the sensitivity. At the same time, the separation ability of MOFs extinguishes interfering substances, to improve the selectivity. The embedding of LGs, makes the optical characteristics of LG@MOFs more adjustable. In addition, the functional groups on the surface of the LG may be the binding sites of the target, which is helpful to improve its sensing performance. Therefore, combining the hybrid characteristics of MOFs and specific optical properties of LGs, for the preparation of LG@MOF composites, with complex pore structures and excellent optical properties, is a promising strategy to fabricate a new generation of fluorescent sensors. To date, an increasing amount of scientific attention has focused on the mechanism and application of these LG@MOF-based nanocomposites. However, there are few reviews on portable colorimetric methods and applications for in situ detection and analysis.

**Figure 1:**
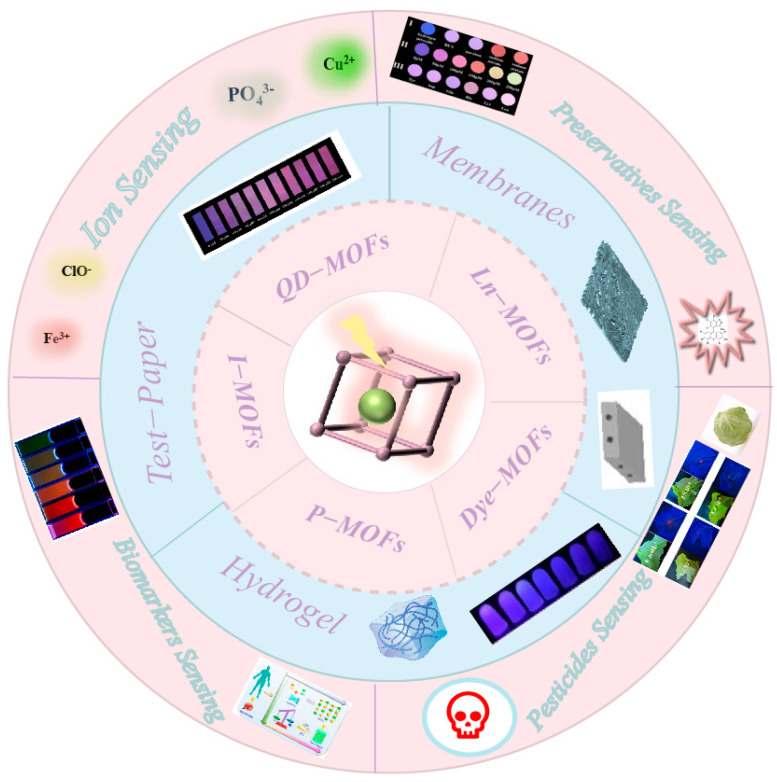
Schematic illustration of the fluorescent characteristics and sensing applications of LG@MOFs. Major types of luminescent guest (LG) that have been encapsulated inside MOF hosts are summarized.

Herein, the construction of LG@MOF sensors and their recent advances in portable fluorescence sensing platforms are reviewed (Figure 1). Lanthanide ions, carbon quantum dots, luminescent complexes, organic dyes, and metal nanoclusters, are encapsulated in MOFs to construct LG@MOF sensors for fluorescence detection of various analytical targets, such as ions, biomarkers, pesticides and preservatives. The prospects for future research in this field and the remaining challenges are also discussed. This review aims to provide an overview of portable fluorescence sensors, focusing mainly on recent research, in the last decade (2012–2022).

## 2. Synthetic Protocols and Design Guidelines

The structure and performance of LG@MOF systems are significantly impacted by the synthetic methods used. The syntheses of this family of LG-encapsulated systems, can currently be classified into two main categories, including “post-synthesis” and “in situ synthesis” techniques.

### 2.1. Post-Synthetic LG Confinement Methods

**Figure 2:**
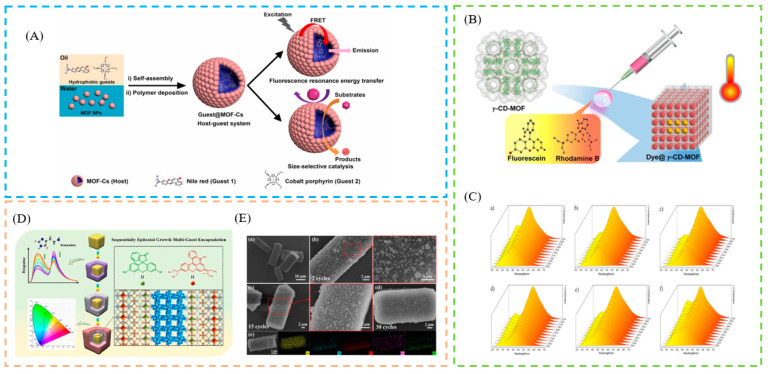
(**A**) Schematic description of the fabrication process of MOF-Cs. (**B**) Schematic description of the fabrication process of dye@γ-CD-MOFs. (**C**) Schematic description of emission maps of spectra recorded in the 253–353 K range. (**D**) Schematic description of the fabrication process of three-dimensional (3D) anionic fluorescence MOFs. (**E**) Schematic description of the SEM images of ZIF-8-on-In-dpda and EDS mapping images of ZIF-8-on-In-dpda (30 cycles). Reproduced with permission from [57,58,59]. Copyright 2020, American Chemical Society; 2022, American Chemical Society; and 2022, Elsevier.

The post-synthetic LG confinement method, refers to the introduction of the LG or precursor by chemical modification, after the synthesis of the MOF material, without destroying the original structure of the MOF. If it is a monomer, the size of the guest must be relatively smaller than the pore window aperture of the MOF host; if it is a precursor, further synthesis steps are necessary to create the appropriate guest [55,56]. The advantage of post-synthetic LG conferment methods, is that the structure of MOFs is modifiable and controllable. For example, Xu et al. first synthesized the MOF (UIO-66), and proposed a universal and flexible strategy to effectively encapsulate hydrophobic guests in MOF capsules (MOF-Cs), without complicated modification. The interaction of the MOF-Cs with encapsulated hydrophobic dyes enhanced the fluorescence resonance energy transfer (FRET) [57] (Figure 2A). In addition, γ-cyclodextrin MOFs (γ-CD-MOFs) have been created and exploited as platforms for dye molecule encapsulation. Peng et al., proposed a simple and effective approach for the preparation of core–shell γ-CD-MOFs, which allows for the effective manipulation of the growth of the γ-CD-MOF shell. The proposed technique allows for more controlled integration of the FL and RhB dyes into the γ-CD-MOFs, thereby improving the efficiency of the established technology [58] (Figure 2B). A 3D anionic fluorescent MOF (In-dpda), with open 1D square channels, has been fabricated by Jiang and co-workers. Sequential epitaxial growth is used to controllably produce classical ZIF-8 with good guest encapsulation ability on the fluorescent core In-dpda, resulting in a ZIF-8-on-In-dpda heterostructure, thanks to the high lattice matching (>97.8%). In addition, by timing the encapsulation of fluorescein with the epitaxial development of ZIF-8 nanoparticles, a sophisticated ratiometric fluorescence sensor F-10c⊂ZIF-8-on-In-dpda is also produced. This sensor has a low detection limit, of 4.7 nM, and high sensitivity to biogenic amines [59] (Figure 2D).

### 2.2. In Situ LG Confinement Methods

**Figure 3:**
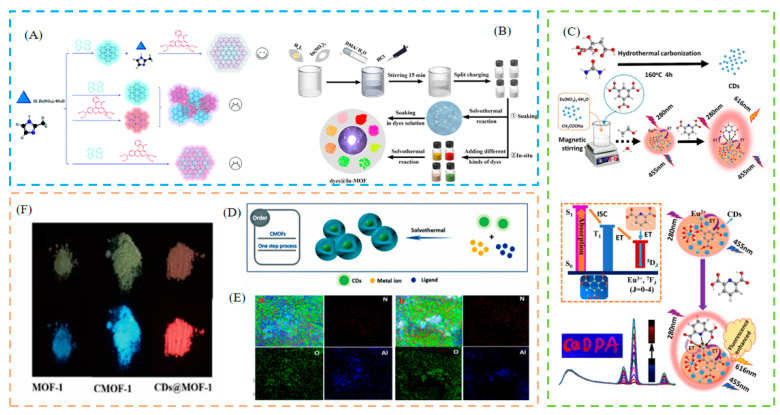
(**A**) Schematic description of the fabrication process of ZIF-8@dye@ZIF-8@dye. (**B**) Schematic description of the fabrication process of single-phase dyes@In-MOF. (**C**) Schematic description of the fabrication process of carbon dots (CDs)-chelated Eu^3+^@metal–organic framework (Eu-MOFs). (**D**) Schematic illustration of growth mechanism for CMOF-1 and CDs@MOF-1. (**E**) Schematic description of mapping images of the CMOF-1 (a) and CMOF-2 (b). (**F**) Photographs of samples exposed to sunlight (up) and under UV light (bottom). Reproduced with permission from [60,61,62,64]. Copyright 2022, Royal Society of Chemistry; 2022, American Chemical Society; 2020, Elsevier; and 2019, Elsevier.

The in situ nanoconfinement approach may also be used to fabricate LG@MOF systems. This packaging method can be simply described as directly combining with the basic building blocks of an MOF (i.e., metal source and organic connector) before the formation of the MOF structure. If the size of the object is larger than the pore size of the MOF, it can also be successfully encapsulated. Compared with post-synthetic LG confinement methods, the synthesis method is simpler and more convenient. For example, Xia et al. presented a hierarchical MOF-on-MOF confinement technique. By using in situ encapsulation and seed-mediated synthesis to build a multilayer ZIF-8@dye@ZIF-8@dye, two types of size-matched dyes (perylene and rhodamine B) were included within the readily available ZIF-8 [60] (Figure 3A). Xu et al., fabricated a variety of single-phase dyes@In-MOF phosphors in situ, and the study significantly increased dye loading and quantum efficiency, while significantly reducing dye leakage [61] (Figure 3B). Yi et al., used a simple one-pot in situ selective self-assembly synthesis method, to effectively synthesize carbon dot (CD)-chelated Eu-MOFs, as dual-emission ratiometric fluorescence (RF) probes. The prepared RF probe has an efficient self-calibration capability and performs highly sensitive and selective detection [62] (Figure 3C). For RF sensing of water in organic solvents, Yin et al. created a guest-encapsulated MOF, Ru@MIL-NH_2_, containing 2-amino terephthalic acid, AlCl_3_, and Ru(bpy)_3_^2+^, by a simple one-pot method [63]. Ma et al., directly prepared unique MOFs from a one-pot hydrothermal reaction, using CDs as the skeletal center. By combining the prepared samples, phosphors with brilliant white light can be produced, which can be used in white light-emitting devices [64] (Figure 3D).

## 3. Encapsulation of Different Luminescent Guests in the MOFs

Given the extreme tunability of MOF structures, a similar diversity in luminous guest types is expected. In general, MOFs can aid in enhancing and improving the luminescence performance of the encapsulated guests for the majority of guest types, primarily by: (1) reducing the ACQ effect and enabling many guests which can only fluoresce in solution to achieve solid-state luminescence, (2) imposing a caging effect on the guests, thereby reducing the non-radiative decay of the guests and increasing their lifetime (τ) and quantum yield (Φ), and (3) there are several MOFs, including ZIF-8, UiO-66, and ZIF-71, which have good thermal and water stability. By using these stable MOF hosts as “shields”, the trapped guests can be shielded, made more stable, and their range of applications extended (Section 6). Organic dyes, metal ions, metal complexes, and nanoclusters, quantum dots, and inorganic–organic (hybrid) perovskites, are the main categories of LGs currently being explored in LG@MOF. The following sections give some current examples of LGs in MOFs.

### 3.1. Lanthanide Ions

Lanthanide metal ions exhibit distinct luminescence characteristics such as a narrow emission peak, large Stokes shift, high fluorescence intensity, and long lifetime, etc. In recent decades, research on lanthanide metal ions has grown exponentially, worldwide. Researchers have mostly studied MOFs doped with lanthanide metal ions, in addition to MOF material built with lanthanide metal ions as the atomic center [65,66]. Wang et al., fabricated dual-emission Eu^3+^@MOFs, by encapsulating Eu^3+^ ions in an MOF host, as a multi-target and self-calibrating probe for the detection of Fe^3+^ and Cr (VI) ions [67]. Due to its wide pores and excellent luminescence capabilities, Liang et al. used Eu^3+^@MOF as a luminescent probe for highly selective Cu^2+^ detection, which is simple and intuitive, by changing the emission color (from red to blue) [68]. Xiao et al., synthesized the Eu^3+^@Mn-MOF with a stable structure and dual emission fluorescence properties, using the post-synthetic modification (PSM) strategy, with emission peaks derived from ligands and Eu^3+^ characteristic emission (antenna effects), enabling fluorescence color transitions between acidic and alkaline solutions, as pH-adjusted toning sensors [69]. Therefore, Eu^3+^@Mn-MOF can be designed as a “histidine-sensitive” fluorescent probe, with the advantages of low detection limit, high sensitivity, and short response time, which has potential applications in the field of biological detection. Luo and co-workers designed Eu^3+^@MOF-253 with exceptional selectivity and high sensitivity (LOD, 0.66 μM) for Cu^2+^ ions in an aqueous solution, due to great suppression of the Eu^3+^ luminescence [70]. Similarly, by fluorescence quenching of Eu^3+^ and MOF over other metal ions, Eu^3+^@MIL-124 was created as a highly sensitive and selective probe for the detection of Fe^3+^ (LOD, 0.28 M) and Fe^2+^ ions [71].

In addition, some other lanthanide metal ions are used to dope MOFs. For example, Ji et al., prepared a fluorescence-functionalized Tb^3+^@Zn-MOF, which not only exhibited excellent chemical stability but also showed high Tb^3+^ emission. Tb^3+^@Zn-MOF was a sensitive luminescence platform with a fast response time of 10 s and a low LOD of 0.1 ppm, for the reversible detection of PO_4_^3−^ ions in aqueous and living cell buffers [72]. Tb^3+^ ions are encapsulated in classical MOFs with uncoordinated N atoms in the pores. Wu et al., prepared Tb^3+^@In-MOF as a food preservative sensor and water scavenger for NO^2−^, demonstrating a highly sensitive ability to detect NO^2−^ in real water samples [73]. Wang et al., prepared a Tb^3+^@Cd-MOF, which exhibited good luminescence and caused a significant quenching effect in the light emission of Tb^3+^ upon the addition of Cr^3+^, and used it as a highly sensitive and selective probe for the detection of Cr^3+^ [74]. These new lanthanide MOFs have many applications in biosensing, imaging, and environmental analysis.

### 3.2. Quantum Dots

Quantum dots (QDs) are 2–10 nanometer semiconductor nanocrystals, with a broad absorption band, a narrow and symmetric emission band, and photobleaching stability. However, QDs can agglomerate after precipitation and drying [75]. A large number of studies have reported that integrating QDs with porous materials such as MOFs, can effectively inhibit electron transitions and holes and improve their stability [76]. By encapsulating polyethylene glycol (PEG)-capped ZnS QDs in ZIF-67 at room temperature, Asadi et al. developed a brand new MOF-based host–guest hybrid system, that enabled highly sensitive and precise detection of Cu (II) ions in water samples [77]. The resulting QDs/CDs@ZIF-8 composite possessed the advantages of an RF sensor, as well as the ability to firmly adsorb target analytes. The composite was used to detect Cu^2+^ ions, and showed great stability and dispersibility in an aqueous solution [78]. Furthermore, by encapsulating CDs and mercapto acetic acid-modified CdTe QDs in situ in MOFs, Yi et al. successfully synthesized a novel dual-emission RF fluorescent probe CDs/QDs@ZIF-8 for the precise and highly sensitive continuous detection of Pb^2+^ (turn-off) and PO_4_^3−^ (turn-on) in biological samples. When Pb^2+^ was added, the fluorescence of CDs/QDs@ZIF-8 changed from red to blue; however, when PO_4_^3−^ was added, the Pb^2+^ on the surface of the material was removed and the fluorescence of the probe was restored [79].

In addition, carbon dots (CDs) is another luminescent ligand that can be encapsulated in MOF. Nitrogen-doped CDs can self-assemble into well-defined spherical nanocomposites (CD@ZIF-CuNC) by physical adsorption. H_2_O_2_ strongly reduces the fluorescence of CD@ZIF-CuNC at 620 nm after 1 min, while it has minimal effect on the emission at 460 nm. As a result, ratiometric quantitation of H_2_O_2_ was achieved using 620 nm fluorescence as the report signal and 460 nm fluorescence as the reference signal [80]. For the detection of Cu^2+^, CDs were encapsulated in Eu-DPA MOFs by Hao et al. When used as the detection and reference parts, respectively, the fluorescence of the Eu-DPA MOFs decreased in the presence of Cu^2+^, while the fluorescence of the CDs remained constant, resulting in a ratiometric fluorescence response to Cu^2+^ [81]. Similarly, by encapsulating optically active CDs and Eu^3+^, a novel, extremely fluorescent hybrid Eu^3+^/CDs@MOF-253 based on MOFs, was prepared. The fluorescent-functionalized MOFs possessed high water stability, in addition to retaining the excellent optical properties of CDs and Eu^3+^ to produce dual emissions [82].

### 3.3. Luminescent Complexes

Metal cationic complexes have stable fluorescence signals in water and organic solvents, such as iridium and ruthenium [83]. Their chromophores in MOFs can improve fluorescence performance [84]. Yin et al., used a one-pot method to encapsulate the red fluorescent cation complex Ru(bpy)_3_^2+^ in the MIL-101(Al)-NH_2_ pore, with blue fluorescence emission, with the emission peaks located at 465 nm and 615 nm.

High sensitivity, low detection threshold, wide detection range, quick reaction, robust stability, and recyclable use are all benefits of the Ru@MIL-101(Al)-NH_2_ fluorescence sensor [63]. Zhao et al., encapsulated [Ir(CF_3_-PPY-F_2_)_2_(bpy)]^+^ (green fluorescence) and [Ru(bpy)_3_]^2+^ (red fluorescence) in the blue fluorescence (ME_2_NH_2_) [Zn_2_(L) (H_2_O)]·4DMA. The presence of volatile organic solvents changed the energy transfer efficiency of the MOFs to the two cationic complexes, thereby changing the color of the complexes from blue to white. When a nitroaromatic hydrocarbon (gaseous state) was detected, the color of the complex became significantly darker. By coordinating the red, green, and blue fluorescence, a multidimensional fluorescence probe was constructed, which can detect volatile organic solvents and nitroaromatic hydrocarbons (in gaseous form), and has the advantages of high sensitivity, good selectivity, and low cost [85]. MnO_2_ NSs prevented the release of Ru(bpy)_3_^2+^, which in turn suppressed the effect of Ru(bpy)_3_^2+^ fluorescence. When GSH was added, MnO_2_ was oxidized to Mn^2+^, which again increased the fluorescence of Ru(bpy)_3_^2+^-UiO-66. Furthermore, with an LOD of 0.28 M, MnO_2_ NS@Ru(bpy)_3_^2+^-UiO-66 can be used to detect GSH in cancer cells [86].

### 3.4. Organic Dyes

Recently, fluorescent dyes have been favored as a unique chromophore, due to their properties such as cheap, accessible high photoluminescence quantum yield, stable optical performance, low toxicity, excellent biocompatibility, wide emission wavelength range, and intrinsic selectivity for the analyte [87]. Organic dyes with superior fluorescence performance are often used as a chromophore to regulate fluorescence, such as cyanine, rhodamine, fluorescein, EY, coumarin, methylene blue, 4-(p-dimethylene styrene)-1-methylpyridine (DMASM) [88]. The introduction of fluorescent dyes into the pores of MOFs can inhibit the aggregation-induced quenching effect (ACQ) in the solid state [89,90], and its superior optical properties are therefore activated. For example, Feng et al. prepared HCAA@UiO-66, which displayed robust fluorescence emission as well as a highly selective fluorescence response to Fe^3+^ ions. UiO-66’s crystalline or porous structure is unaffected by the presence of HCAA in its pores. The fluorescence quenching was brought on by the interaction of Fe^3+^ ions with HCAA@UiO-66 and the ACQ [91]. Li et al., rationally designed fluorescent probes for the determination of inorganic phosphate (Pi). This probe was constructed by loading uranine into the pores of ZIF-8, during which aggregation-induced quenching occurred. In the presence of Pi, due to the decomposition of ZIF-8, uranine was released, resulting in increased fluorescence. The probe was easily prepared at room temperature by a one-step method, with a fast response of 3 min and a low LOD of 0.2 μM [92]. The Rh110@MOF-801 fluorescent probe was prepared by Huang et al., using a one-pot synthesis technique, and was effectively used for nitrite detection. This approach makes full use of rhodamine 110 (Rh110) molecules and porous MOFs, which not only eliminates the disadvantages of hydrophobic dye molecules but also enhances their fluorescent properties. The binding between nitrite and fluorescence recognition sites is additionally promoted by the open structure and strong stability of porous MOFs, which further enhances the detection capability. It can be used for quantitative detection of nitrite content in tap water, as its fluorescence intensity is linearly negatively related to nitrite concentration in the range of 2–7 μM and the LOD is as low as 0.2 μM [93].

### 3.5. Metal Nanoclusters

Fluorescent metal nanoclusters are a type of nanomaterial consisting of tens to hundreds of metal atoms, between a single metal atom and larger metal nanoparticles. The size of metal nanoclusters is close to the Fermi wavelength (<2 nm) [94]. Compared with larger nanoparticles, metal nanoclusters have more unique physical and chemical properties, thus showing excellent optical properties, such as adjustable fluorescence emission, large Stokes shift, and high fluorescence stability. Compared with traditional fluorescent dyes and quantum dots, the toxicity of fluorescent metal nanoclusters is greatly reduced, and the biocompatibility is significantly improved, but the quantum yield of metal NCs is low [95,96,97]. Therefore, some studies have been reported to improve the quantum yield. Some MOF encapsulation-based metal nanoclusters can improve fluorescence performance. This can be achieved by confining the metal nanoclusters within the MOF host, to generate a core–shell composite. Importantly, the immobilization of metal nanoclusters in MOF hosts can inhibit the clustering and aggregation processes that reduce luminescence efficiency. For example, it has been observed that the properties of CuNCs improved when they were confined within the framework of an MOF host.

Due to the restrictive structure of MOFs, the stability of CuNCs enclosed in an MOF was extended from three days to three months, and the fluorescence intensity was raised by roughly 35 times. By centrifuging in an aqueous solution, the CuNCs@GSH/MOF-5 composites were extremely stable and recyclable as made [98]. Similarly, an in situ encapsulation method of CuNCs for ZIF-8 was shown to increase the stability and emission intensity of CuNCs. The blue emission intensity was reduced by 1.0 μM, to 10.0 μM, by adding tetracycline to the CuNCs@ZIF-8 solution, and the LOD was 0.30 μM [99]. Chen and co-workers prepared AuNCs@zinc glutamate MOFs (AuNCs@ZnGlu-MOFs), using a unique one-pot method. Its good optical properties suggest that it could be used as a selective and sensitive fluorescent probe for the detection of H_2_O_2_ and H_2_O_2_-related analytes [100].

A novel dual-emission fluorescence sensor, CuNCs@Tb@UiO-66-(COOH)_2_, for the detection of Cu^2+^ was prepared by encapsulating Tb(III) and glutathione-stabilized CuNCs in UiO-66-(COOH)_2_, in a one-pot reaction. The fluorescence intensity of Tb^3+^ decreased dramatically when Cu^2+^ was added to this ratiometric sensor, but CuNCs showed high stability and an obvious color change [101]. Furthermore, Jalili et al. reported a ratiometric fluorescent probe based on in situ integration of gold nanoclusters (AuNCs) and green emitting carbon dots (gCDs) in ZIF-8, for cephalexin (CFX) measurement. CFX selectively quenches the fluorescence of AuNCs (630 nm) at doses ranging from 0.1 to 6 ng/mL, with a low LOD of 0.04 ng/mL [102]. Given this promise, there is growing research focusing on the development of metal NCs@MOF systems.

## 4. Portable Detection Device Smartphone Platform

To date, a large number of MOF fluorescence sensors have been reported, to detect target analytes. However, for most of the reported fluorescent sensors, the fluorescence emission signal is relatively single and susceptible to environmental factors, which cannot meet the needs of rapid detection for field visualization. To address this problem, visual portable detection, based on LG@MOFs, with a smartphone, may be an effective solution. Smartphones can more accurately detect the color difference on each pixel, by calculating RGB (red, green, and blue) values. Compared with traditional methods, the portable sensing platform based on smartphones has obvious advantages such as low cost, real-time response, easy operation, accurate measurement, and portability, and has been widely used in clinical diagnosis [103,104,105,106], environmental monitoring [107,108,109,110] and food analysis [111,112,113]. Researchers have been actively engaged in fluorescence measurements compatible with smartphones, to develop on-site sensing platforms for quantitative detection.

### 4.1. Surface Plasmon Coupled Emission (SPCE) Platform

This departure from conventional detection systems towards handheld devices has been pursued on account of the advantages of the smartphone in terms of easy transportability, unparalleled data acquisition ability, superior computing, and ever-refining premium quality camera technologies [6,114,115,116]. The SPCE platform, is a prism coupling technique, where the fluorescence is coupled to the surface plasmon polaritons (SPPs) of the metal thin film, assisting in the realization of >50% signal collection efficiency. It has been observed that the developed SPCE platforms can be synergized with the ubiquitously available smartphone-based detection systems. For example, Bhaskar and co-workers present dielectric-based nanostructured carbon florets (NCFs) for 1000-fold fluorescence enhancements, to detect perindopril erbumine at a single molecule level on a smartphone-enabled visual. It is observed that there is an excellent correlation between the luminosity values (smartphone) and the SPCE enhancements, thereby establishing the effectiveness of smartphone-based sensing in a reliable and reproducible manner [117]. Bhaskar et al., demonstrate the effect of HRI dielectric TiCN NCs, along with GO, in spacer nanointerface as SPCE substrates, with unprecedented >800-fold emission enhancements. The SPCE obtained for the different samples was captured using a smartphone, and the images were processed using the color grab app to obtain the luminosity values [118].

### 4.2. Test Paper

**Figure 4:**
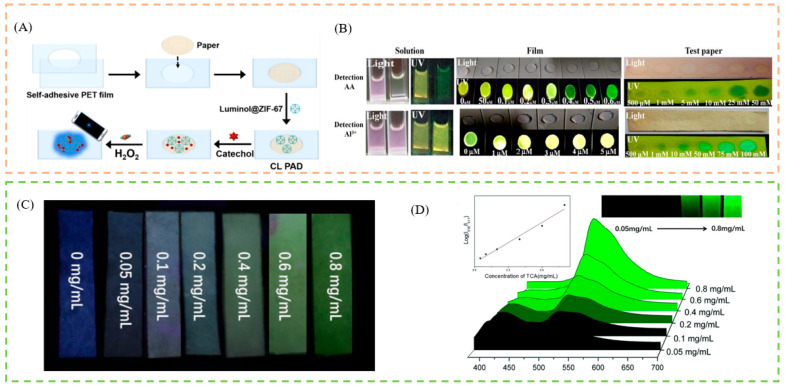
(**A**) Schematic description of cobalt–imidazole metal–organic framework loaded with luminol, for paper-based chemiluminescence detection of catechol with the use of a smartphone. (**B**) Schematic illustration of application for AA and Al^3+^ detection by FL@UiO-67 solution, FL@UiO-67 film, FL@UiO-67 test paper. (**C**) Schematic illustration of application for TCA detection by test strips. (**D**) Schematic illustration of emission spectra of the FS@1 in the presence of different concentrations of TCA in real urine. Reproduced with permission from [121,122,123]. Copyright 2021, Springer; 2021, Elsevier; and 2019, Royal Society of Chemistry.

With the shortage of resources today, portable online detection methods based on visual test strips and smartphones are very popular, and related dye-coated sealed test strip probes have also been reported [119,120]. Fluorescent paper chips, consisting of fluorescent probes and paper strips with outstanding characteristics such as low cost, easy fabrication, storage, and transportation, are ideal candidates for integration with smartphones, for point-of-care (POC) detection, making the sensing process more intuitive and convenient. Li et al., developed a dipstick-based chemiluminescence (CL) imaging detection method to identify catechol, using a smartphone as a portable detector. As the catechol content increased, the CL emission decreased accordingly. ΔGray values were calculated by subtracting the gray value obtained in the absence of catechol, from the gray value measured in the presence of catechol. The concentration of catechol was calculated from the Δgray value. In the range of 5 to 100 mg/L, the gray values of the light spots showed a strong linear relationship with the logarithm of the catechol concentration [121] (Figure 4A). Yang et al., prepared a successful test paper for visual detection of Al^3+^. Under UV light, the fluorescence of the test paper changed dramatically as the quantity of Al^3+^ increased, which could be used to detect Al^3+^ qualitatively [122] (Figure 4B). Wang and co-workers prepared a portable paper-based probe for the easy detection of TCA, which exhibited a unique fluorescence shift. The FS@1 test strip (1 × 3 cm) was used by dipping it in human urine samples for 1 min and drying it at room temperature. The fluorescence images of these strips were captured using 365 nm UV irradiation. At increasing TCA content, the fluorescence emission intensity of the test strips increased. The colors of different intensities could easily be distinguished with the naked eye, allowing them to assess the level of intoxication caused by TCE exposure [123] (Figure 4C). Furthermore, Fu et al., created CDs@MOF(Eu) to detect doxycycline, by quenching the blue light emission of CDs while enhancing the red light emission of MOF (Eu). In light of this result, a more convenient test paper was first used as a novel instrument for doxycycline detection, whose color changed from blue–purple to red when exposed to 365 nm UV irradiation [124]. Wang et al., designed Eu^3+^@CdK-MOF as an excellent fluorescence sensor for the detection of ornidazole (ODZ), as well as a portable ODZ test paper [125]. A fluorescence color change was observed when the test paper was infiltrated with different antibiotics and different concentrations of ODZ. The Eu^3+^@CdK-MOF test paper emits bright red fluorescence under UV light at 254 nm in the presence of other antibiotics. The color of the test paper changed from red to blue as the ODZ concentration increased from 0 to 1 mmol/L. As a result, the portable ODZ test paper, which can be seen with the naked eye, has significant practical value. Zhou et al., designed a novel dual-emissive fluorescence nanoplatform for ATP sensing, based on a red emissive europium metal–organic framework (Eu-MOF) and blue emissive gold nanoclusters (AuNCs). Moreover, a fluorescent paper-based sensor was fabricated with the ratiometric ATP probes, which enabled easy-to-use and visual detection of ATP in serum samples with a smartphone [126]. Kong et al., prepared a BSA@AuNCs@MOF to detect heparin and chondroitin sulfate based on smartphones. The dual-emission reverse change ratio fluorescence nanoplatforms possessed variations in the emission intensity ratio at two different wavelengths, and the corresponding green-to-red fluorescent color changes [127].

### 4.3. Membranes

Because of their affinity for nanoparticles, high mechanical strength, and non-toxicity, biopolymers have been widely used as carrier materials. A growing number of reports have reported that MOF-based cellulose composites exhibit stronger properties in both electrochemical and contaminant removal. For example, Zhang et al. used a simple one-step solvothermal approach to prepare two electroactive polyoxometalate (POM)-based MOF films (POMOFs), grown in situ on carbon cloth (NENU-3/CC and NENU-5/CC). As a result, two types of film electrodes show enhanced electrocatalytic activity for bromate reduction in an acidic solution. They can be used as electrochemical sensors for bromate detection [128]. Wang et al., prepared a responsive dye@bio-MOF-1 film, that can be used as a dual-emitting platform for enhanced detection of various nitro-explosives, including nitroalkanes, nitramines, and nitrate esters [129]. When the concentration was reduced to 1 µM, the film still showed a clear fluorescence response after 1 min of immersion in explosive solutions, and the response was time-dependent. This result indicated that the detection of explosives was fast and sensitive. Wang and co-workers synthesized high-quality continuous copper network-supported MOF-5 membranes for the first time, using vapor diffusion of an organic amine. The rate of organic amine diffusion and the reaction temperature has a direct effect on the quality of the membranes produced. In addition, a laser dye is effectively entrapped in such membranes in CHCl_3_. The dye-loaded MOF-5 membranes can be used in optics, particularly in laser systems [130].

### 4.4. Hydrogel

**Figure 5:**
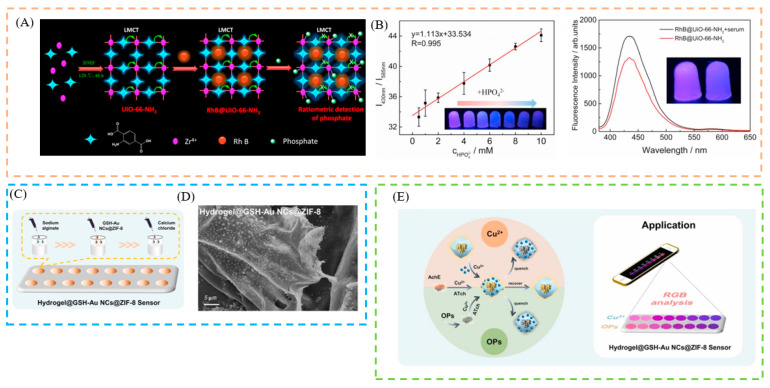
(**A**) Schematic illustration of the synthesis of RhB@UiO-66-NH_2_ and the possible mechanism for ratiometric detection of phosphate. (**B**) Schematic illustration of the linear calibration curve of RhB@UiO-66-NH_2_ agarose hydrogels and phosphate concentration in the range of 0.5–10 mM. (**C**) Schematic illustration of synthesis of hydrogel@GSH-Au NCs@ZIF-8. (**D**) SEM images of hydrogel@GSH-Au NCs@ZIF-8. ((**E**), left) Fluorescent sensors for Cu^2+^ and OPs detection. ((**E**), right) Hydrogel-based sensor, based on GSH-Au NCs@ZIF-8 integrated with smartphone platform, for on-site detection. Reproduced with permission from [131,132]. Copyright 2022, Elsevier; and 2022, Elsevier.

In addition to fiber membrane and test paper-based portable detection, the developed hydrogel method has also attracted extensive attention. A three-dimensional network structure, using water as a dispersing medium, is known as a hydrogel. It is soft, dimensionally stable, and has a high water absorption capacity. In addition, hydrogels have strong biocompatibility and biodegradability and are widely used in biomedicine. For example, RhB was added to the stable Zr-based MOF UiO-66-NH_2_, to form a nanocomposite by Gao et al. It is particularly suitable for accurate phosphate detection in challenging samples, such as human serum. To facilitate the visual detection of phosphate in human serum, this nanocomposite was encapsulated in agarose hydrogels [131] (Figure 5B). For the detection of Cu^2+^, Wei et al. prepared GSH-Au NCs@ZIF-8 by encapsulating GSH-Au NCs with an AIE effect in MOFs. In addition, due to its ability to inhibit the activity of AChE, thiocholine (Tch), the hydrolysis product of acetylthiocholine (ATch) by acetylcholinesterase (AchE), could coordinate with Cu^2+^ through sulfhydryl groups (-SH), resulting in a significant fluorescence recovery. In addition, the combination of a hydrogel sensor with a smartphone sensing platform was investigated, to enable device-free, visual, and quantitative monitoring of Cu^2+^ and OPs [132] (Figure 5C).

## 5. Sensing Applications

With the enrichment of human material wealth, health, environment, and food safety have become hot issues of global concern. The human body, the environment, and food are all inseparable systems. Toxic and harmful substances acting on any part of this system will inevitably endanger human health and well-being. So far, effective monitoring of toxic and harmful substances requires close integration of multiple disciplines and technologies. Making full use of the integration advantages of new technologies, to achieve efficient monitoring of toxic and harmful substances, has aroused the widespread interest among scientific and technological researchers. Toxic and harmful substances released from industrial and agricultural production activities seriously damage human health. Therefore, it is of great significance to establish accurate detection methods for toxic and harmful substances in human body fluids, the environment, and in food. In addition, the lack of biological ions in the human body is closely related to the occurrence and development of many diseases (Table 1 and Table 2). Efficient monitoring technology can achieve disease prevention and early detection. It provides a very important prospect for clinical diagnosis. Therefore, detection technology with high sensitivity, specificity, simplicity, rapidity, accuracy, recyclability, portability, visualization, and intelligence should be developed.

### 5.1. Sensing of Ions

**Figure 6:**
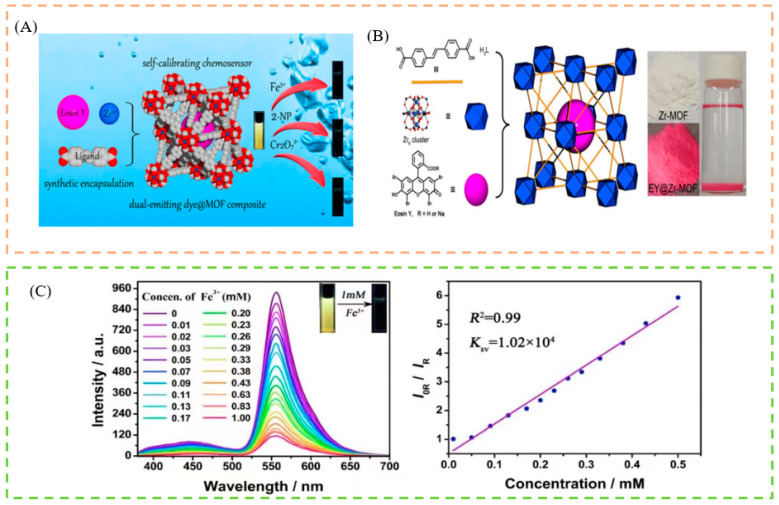
(**A**) Schematic illustration of the detection of Fe^3+^, Cr_2_O_7_^2−^, and 2-nitrophenol, by the luminescent sensor. (**B**) Schematic illustration of fabrication of the EY@Zr-MOF composite via the in situ synthetic encapsulation method. (**C**) Schematic illustration of the concentration-dependent fluorescent emission spectra upon the different contents of Fe^3+^ in aqueous solutions. Reproduced with permission from [137]. Copyright 2019, American Chemical Society.

Metal ions are ubiquitous in our lives and the environment. An appropriate amount of ions is beneficial to human health and the sustainable development of the environment. However, when the ion concentration exceeds a certain standard, it will cause many serious hazards, such as environmental pollution, ecological imbalance, and various physiological diseases. Iron is a fairly abundant element, accounting for 4.1% of all elements on the Earth. However, an excess of iron will cause environmental problems, such as soil pollution and water pollution. Iron is an essential trace element for the human body and is required for the production of many enzymes, e.g., myoglobin, and haemoglobin. It is widely distributed in the blood, muscle tissue, liver, bone marrow, and other tissues, and organs. When an organism takes in too much iron or lacks iron, it will affect the health of people, animals, and plants. Accurate detection of metal ions is therefore essential for maintaining the environment and preventing disease. Fluorescence detection, particularly visual detection, has a fast response, high selectivity and sensitivity, and ease of operation, compared to conventional analytical methods for detecting metal ions, such as atomic absorption spectroscopy, inductively coupled plasma mass spectrometry, and electrochemistry [133,134,135]. Tao et al., prepared Eu^3+^/CDs@MOF, a novel composite material that emits intense red light at room temperature and shows good fluorescence sensing properties for Fe^3+^ ions, and the color change can be observed by the naked eye [136]. Li et al., fabricated a dual-emitting composite by using a synthetic encapsulation technique to combine a fluorescent dye, Eosin Y (EY), with a Zr-MOF, which was used as a self-calibrated luminescent sensor for selectively detecting Fe^3+^. The fluorescence phenomenon gradually decreases (yellow→colorless) [137] (Figure 6A). Liu et al., built a dual luminescent RhB@Mn-MOF material, to selectively detect Fe (III) [138]. Cationic acriflavine dye, as a fluorescent dye molecule, was selected to be introduced into the pores of anionic bio-MOF-1 through ion exchange. The resulting Acf@bio-MOF-1 serves as a fluorescence sensing probe for the specific and sensitive detection of Fe^3+^, which showed a distinct color change (blue to green) [139]. By in situ encapsulation of luminescent RhB molecules into a blue-emitting Zr-MOF, Zhang and co-workers created a series of dye@MOF composites, which have tunable dual-emissive properties, one of which can be used as self-calibrating platform to detect Fe^3+^ in water [140]. The color shift can be seen with the naked eye (red to colorless). Such a robust and reliable smartphone-based detection system, assists in cost-effective and portable analyte quantification routes. Furthermore, to re-emphasize the utility of the colorimetric method for the development of biosensing frameworks, a comprehensive analysis of the literature is presented in Table 3. Here, the performance of different techniques for the detection of Fe^3+^, and its comparison with the fluorescence, is tabulated, where we observed that only fluorescence has a better colorimetric performance.

**Figure 7:**
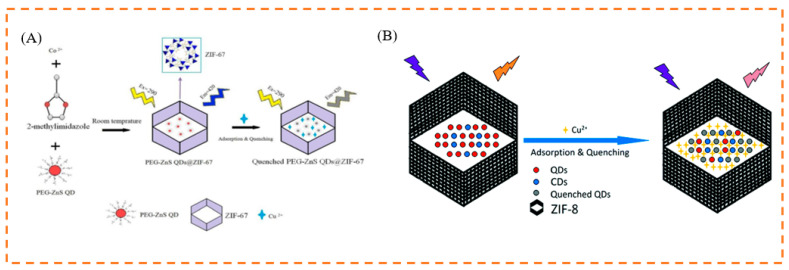
(**A**) Schematic illustration of the process for sensing Cu^2+^ based on fluorescent PEG-ZnS nanohybrids. (**B**) Schematic illustration of the process for sensing Cu^2+^ based on fluorescent QDs/CDs@ZIF-8 composite. Reproduced with permission from [77,78]. Copyright 2019, Elsevier; and 2017, Royal Society of Chemistry.

In addition, the Cu (II) ion, one of the metal ions in biological media, is important in living systems. However, it is toxic to the human body when present in high concentrations, and can lead to neurodegenerative diseases [141]. Therefore, it is essential to provide a sensitive and focused fluorescent chemosensor for rapid on-site detection of Cu^2+^. For example, Asadi et al. developed a brand new MOF-based host–guest hybrid system, by encapsulating polyethylene glycol (PEG)-capped ZnS quantum dots (QDs) into ZIF-67. The resulting PEG-ZnS nanohybrids benefited from the accumulation effect of ZIF-67, as well as the FL sensitivity and selectivity of ZnS QDs towards Cu^2+^. The probe could detect Cu^2+^ over a wide concentration range, from 3 to 500 nM, with an LOD as low as 0.96 nM [77] (Figure 7A). The PEG-ZnS QD@ZIF-67 sensor was then used to detect copper (II) ions in tap water. The fluorescence studies of QDs@MOF showed a dramatic improvement in the fluorescence quantum yield compared to the FL sensor based on QDs without MOF. Similarly, Ma et al. synthesized QDs/CDs@ZIF-8 to detect Cu^2+^ ions with the color change from orange to pink, which not only possessed the advantages of an RF sensor, but also accumulated target analytes strongly, due to the adsorption ability of MOFs [78] (Figure 7B).

**Figure 8:**
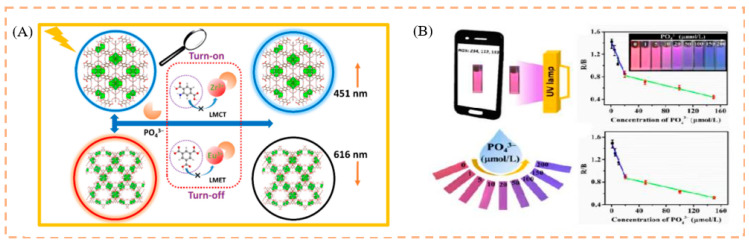
(**A**) Schematic illustration of the detection of phosphate by the RF probe. (**B**) Schematic diagram of phosphate detection by a smartphone. Reproduced with permission from [144]. Copyright 2021, Elsevier.

The phosphate radical (PO_4_^3−^) is intimately associated with many critical physiological and pathological processes, as a vital metabolic and genetic substance in living systems. However, increased PO_4_^3−^ concentrations are linked to cardiovascular disease and acute renal failure. While numerous analytical methodologies for identifying PO_4_^3−^ have been published, their use and promotion in quick and real-time detection are severely limited [142,143]. As a result, the development of a visualization approach for quick, low-cost, and tiny equipment, has a very high practical application potential for on-site recognition of PO_4_^3−^. For example, Yi et al. fabricated a hybrid fluorescent NH_2_ and Eu^3+^@MOF-808, to detect PO_4_^3−^. The detecting platform’s fluorescence response to PO_4_^3−^ showed a definite color shift (red→pink→blue). More importantly, the probing solution and test paper from the embedded smartphone can be successfully translated into digital values via RGB channels and used to show the semiquantitative identification of PO_4_^3−^ [144] (Figure 8). The use of a smartphone with the developed probe and fluorescent test paper platform, provides a unique approach for intelligent online identification of relevant targets in biological and environmental samples.

Hypochlorite (ClO^−^), a potent bactericide and disinfectant, is widely used in residential drinking water disinfection, bleaching, and wastewater treatment [145]. It is difficult to eliminate bacteria and viruses if insufficient ClO^−^ is added to the water [146]. However, too much residual ClO^−^ will result in a large number of hazardous by-products in the water [147]. To detect ClO^−^, Xiong et al. prepared an Acriflavine@lanthanide metal–organic framework (Acr@Eu(BTEC)), by covalently fusing an amino-rich dye (Acr) and a carboxyl-rich Eu(BTEC), with a post-synthesis method [148]. The resulting fluorescence sensor has two emission centers, one from Acr and one from Eu(BTEC). While the invariant red emission from Eu^3+^ served as a reference signal, the robust green fluorescence from Acr was strongly quenched in the presence of ClO^−^. Furthermore, the distinct fluorescence color change, from green to orange to red, allows visual detection of ClO^−^ with the naked eye. This technique shows good performance in real conditions, suggesting that it has great potential for water quality monitoring. Therefore, this visual encapsulation strategy can be used to sensitively detect inorganic ions.

### 5.2. Sensing of Biomarkers

**Figure 9:**
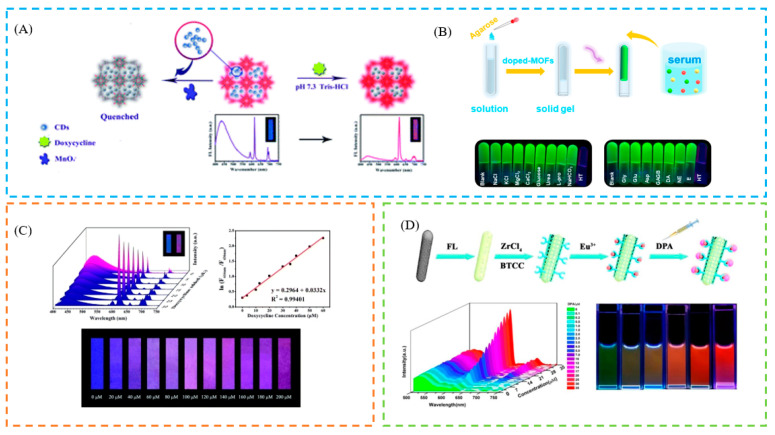
(**A**) Schematic illustration of the process for sensing doxycycline, based on fluorescent CDs@MOF. (**B**) Schematic illustration of the mechanism for BR detection by Tb^3+^@MOF-808 and photographs of the fluorescent filter paper strips sprayed with various analytes. (**C**) The color change of test paper with the concentration of doxycycline. (**D**) Schematic illustration of the preparation of a Pal@FL@UiO-66-(COOH)_2_-Eu nanoprobe and the multicolor fluorescence detection of DPA and the fluorescence images of Pal@FL@UiO-66-(COOH)_2_-Eu in the presence of DPA. Reproduced with permission from [124,153,154]. Copyright 2018, Royal Society of Chemistry; 2022, Elsevier; and 2020, Elsevier.

In the field of bioanalytical and clinical diagnostics, biomarkers associated with diseases have attracted increasing attention [149,150,151]. Therefore, it is crucial to develop a reliable biosensing technique for biomarker identification. Using a concerted post-synthetic modification technique, Yi et al.,created a new water-stable Tb^3+^@MOF-808 for the identification of aberrant bilirubin (BR) in serum and urine. Importantly, the developed fluorescent probe was successfully applied to the preparation of portable BR test paper [152]. This probe has realized portable colorimetric detection. Fu et al., incorporated carbon dots (CDs) into a luminescent MOF(Eu), which showed remarkable doxycycline selectivity and sensitivity. As a more practical test paper, that changed color from blue–violet to red when exposed to 365 nm UV radiation, it was first used as a new tool for doxycycline detection [124] (Figure 9A). Song et al., successfully constructed a new fluorescent material doped with NOTT-220 for ratiometric fluorescence detection of the carcinoid biomarker HT in serum. The energy transfer from the ligand to Tb^3+^ was inhibited in the presence of HT, quenching the fluorescence emission at 545 nm while enhancing it at 350 nm. Meanwhile, a promising method for visual HT assay was developed using the fabricated portable fluorescent hydrogel as a multifunctional platform for simple, rapid, and real-time HT sensing, with the color change being visible to the naked eye (green→colorless) [153] (Figure 9B). This study not only created a novel method for ratiometric fluorescence and visual detection of HT, but also expands the literature for future clinical early disease detection. Jia et al., functionalized the palygorskite (Pal) hybrid of MOFs as a new multicolor fluorescent probe for the detection of the biomarker dipicolinic acid in bacterial spores (DPA), which showed a pronounced color change (green→red). The results showed that the LOD of this multicolor fluorescent probe was as low as 9.3 nM, which is significantly lower than the concentration of anthrax spores that can infect a human body (60 μM). In addition, a wide linear range, of 0 to 35 μM, was achieved [154] (Figure 9C). Sun et al., developed a practical approach method for NMF detection with luminescent Eu^3+^-functionalized MOFs (Eu(III)@MOF-1). Even in the presence of other chemical constituents of urine, a significant increase in the luminescence of Eu(III)@MOF-1 can only be achieved by NMF [155]. Moreover, a portable test card is also produced for rapid detection (blue→pink). To monitor the poisoning of DMF contact, the NMF sensing method can serve as a promising visual application.

### 5.3. Sensing of Pesticides

**Figure 10:**
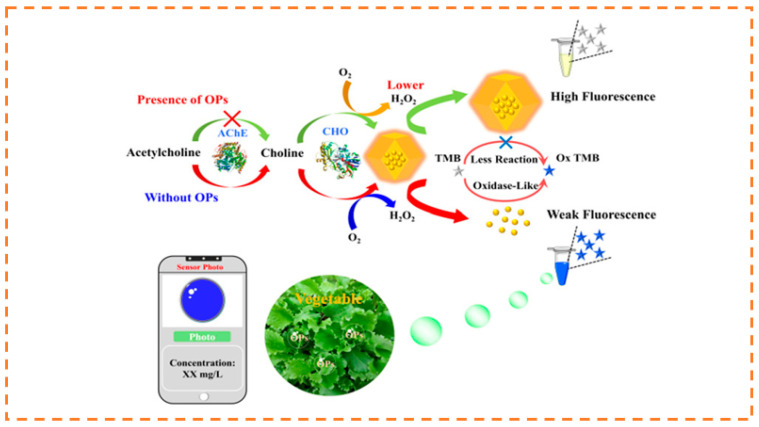
Schematic illustration of the mechanism for the detection of OPs. Reproduced with permission from [158]. Copyright 2021, American Chemical Society.

The abuse of pesticides has caused serious environmental pollution, so the development of efficient, simple, and sensitive pesticide detection technology is a research focus in the field of environmental science. Due to its high sensitivity, low detection limit, simple operation, and low cost, fluorescence sensing detection has emerged as a development direction for rapid quantitative detection of pesticides. For example, Wei et al. prepared a series of eosin Y (EY)-embedded Zr-MOFs, using the synthetic encapsulation approach, for pesticide detection, where EY@Zr-MOF achieved the selective detection of nitenpyram with an obvious color change (blue→orange) [156]. Liu et al., prepared two europium-post-doped MOFs Eu^3+^@1 and Eu^3+^@2, which can achieve the quantitative detection of pesticides with high sensitivity and selectivity in an aqueous solution, exhibiting a color change (blue→colorless) [157]. Cai et al., encapsulated AuNCs into an MOF, which can cause the aggregation-induced emission (AIE) effect. Based on the dual ability of the enzymolysis product of AChE and choline oxidase (CHO) on AuNCs@ZIF-8 to detect OPs, the coupling of fluorescence and colorimetric signals was realized. A smartphone app was also developed to improve the visualization results and allow real-time monitoring of pesticide contamination. Colorimetric paper strips were used for visual semiquantitative detection (blue→colorless) [158] (Figure 10).

### 5.4. Sensing of Preservatives

In addition, a similar technique can be used to evaluate preservatives. An electron-deficient MOF is used to encapsulate the anionic dye interhydrodiphenol B (PB), resulting in a novel fluorescence “on” behavior, by preventing light-induced electron transfer. For preservatives with different hues and visual (blue–red) quantitative discriminatory effects, PB@UiO-67-CDC-(CH_3_)_2_ exhibits a distinctive fluorescence response that could be used for sensing, switching, and anti-counterfeiting identification [159].

### 5.5. Stability of Sensors

Improved understanding should be sought about the photostability of LG@MOF system-based portable devices subject to UV irradiation and other environmental factors. Information on the long-term durability of LG@MOFs under ambient conditions is particularly lacking in the literature [160], however, the stability of the sensor can be improved by preparing it as a portable device, such as packaging it in a hydrogel. For example, Jia et al., designed an MOF-modified gel (Zn2@ZIF-8@SA) {[Zn(tbia)·H_2_O]·H_2_O}n(Zn_2_) that was portable, had a good stability, compression resilience, and was toxicologically innocuous, by a simple polymerization strategy, with which to detect and remove pesticides from the environment. Indeed, the Zn2@ZIF-8@SA was stable in H_2_O, evaluated up to 30 days, and in harsher environments including various organic solvents (i.e., N,N-dimethylformamide (DMF), acetonitrile (CH_3_CN), ethanol (EtOH)) up to 7 days [161]. Lu et al., prepared CDs@Fe/Zr-MOF based on a smartphone-assisted dual-color ratiometric fluorescence smart gel label-based visual sensing platform. The results show that the fluorescence intensity and basic morphology of hydrogel tags do not change with time (over 6 consecutive days) at different temperatures (−20 to 35 °C), indicating that the smart tags used in this system have good stability [162]. Jia et al., developed a simple and effective strategy for the visual determination of BAs by dual-emissive metal–organic framework (MOF) probes and color-transition hydrogels, to real-time monitor biogenic amines (BAs). Meanwhile, the stability of fluorescent sensory hydrogels was confirmed, by depicting the spoilage degree of shrimp and fish at 4 and 20 °C for 3 days [163]. In addition, Subramaniam and co-workers demonstrated the importance of exploring the sort nano-assemblies synthesized using a lower temperature (−18 °C) gradient for surface-enhanced Raman scattering and associated biosensing applications [164,165]. Following this revelation, in 2022, Ramamurthy and co-workers demonstrated the utility of extremely low temperatures (−80 °C, −150 °C, and −196 °C) for nano-assembly synthesis via cryosoret nano-engineering (CSNE), generating metal, metal-dielectric and metal-graphene oxide-based hybrid nano-assemblies, with tailorable plasmonic hotspots, for the realization of precise nano-assemblies and associated photo-plasmonic hotspots, for applications in biosensing, using the SPCE platform [166].

## 6. Future Scope and Perspectives

It is encouraging that research on LG@MOF materials and their composite systems is being widely addressed. Nano-confinement or encapsulation of LGs within an MOF host is a fundamental shift from the traditional approach to producing LG@MOF materials with inherent luminescence. The literature is rapidly increasing and, so far, a large number of attractive photophysical and photochemical properties have been produced, with interesting possibilities for innovative applications. In this review, we show the construction of LG@MOF materials and their portable detection systems. However, this is accompanied by the shortcoming of restricting the incorporation of smartphone-based detection technologies. Newer materials need to be investigated to overcome the above-mentioned challenges and limitations. A summary of the methodologies and their performance, in terms of merits and demerits of fluorescence spectroscopy, ion chromatography, SERS, SPCE, and the electrochemical method, is tabulated in Table 4. These details are envisaged to assist researchers in the broad domain of fluorescence spectroscopy and biosensors, to further explore nano-engineering and biosensing modalities in this direction. With the increasing number of reported LG@MOF systems and their potential applications, we can discover the challenges surrounding this new field in time for further systematic investigation, from the perspective of basic science and practical applications.

(1) Synthesis: the luminescence properties of LG@MOFs are mainly dependent on the framework structure of the MOF materials and the synergistic effect between the photon units. The main limitation of the framework structure is that the pore size, structure, metal node, and organic ligand properties of MOFs must match the size, surface charge, and inherent properties of the fluorophore. Synergism refers to the relationship between the synergistic effects of different metal nodes such as photon units, organic ligands, and guest molecules. Therefore, the relationship between structure and properties, preparation strategies, host–guest interactions, and synergistic effects of LG@MOFs require further study.
(2) Detection: most detection methods are based on “off” processes, while LG@MOF materials based on “on” processes can improve selectivity and sensitivity. At the same time, most established LG@MOF sensors depend on the change in transmission signal strength from a single transmission center, which may lead to false responses due to the change in external conditions. The ratio fluorescence probe and self-calibration method can eliminate environmental interference and improve detection accuracy. In addition, it can also add an adsorption function and create a new luminous LG@MOF, combining detection and eradication. Due to the complexity of actual samples and the trace level of target pollutants, it is necessary to consider adding functional groups to LG@MOFs, for sample purification and target analyte enrichment.
(3) Sensing mechanisms: the mechanism of fluorescence sensing still needs further investigation and theoretical calculations are needed to better understand the sensing process. The mechanisms proposed by many reported studies on LG@MOFs are either vague or speculative. The application of theoretical methods, such as density functional theory (DFT), should be encouraged for LG@MOF systems. The main challenges lie in modeling the dispersion correction and electronic structure of large-scale systems with spatial constraints, and using DFT to simulate excited-state events. Therefore, the recent development and implementation of a computationally efficient but accurate DFT method, are expected to simulate the structure–property relationship of large LG@MOF systems.
(4) Application: the versatility of such composites and practical applications need further exploration, such as the activity and selectivity for catalyzing tandem reactions, optical imaging for biotherapeutics, diagnostics, and drug delivery characteristics. The use of LG@MOFs with customizable sizes and shapes for targeted drug delivery, living cell sensing, and imaging is also promising. In addition, sensing devices such as test papers and luminescent labs-on-a-chip, should be designed for convenient use, by the combination of the properties of LG@MOFs. Recent advances in high-resolution 3D printing, precision inkjet printing, electrospinning, and lithography, can also be combined, to expand LG@MOF-based applications. We believe that, with continuous research and improvement, a bright future for LG@MOFs in the field of fluorescence detection can be expected.

## 7. Conclusions

In conclusion, this study reported on the development of research into the construction of LG@MOF materials and their portable detection systems. More and more researchers are interested in using LG@MOF for the measurement of ions, biomarkers, pesticides, and preservatives. In addition, portable devices with smartphones are desired for real-time applications in the field. These devices have the potential to meet critical needs for simple, rapid, and accurate testing in remote and resource-constrained settings, and can be used in a variety of detection platforms for monitoring biological phenomena, healthcare, environmental pollution, and food safety.

**Table 1:** Performance of MOF-based fluorescent chemosensors for the detection of small organic molecules.

Targets	MOF	Color Change	Linear Range	LOD	Ref.
1-N	Eu^3+^@MOF-253	red→green		7 μg/mL	[167]
OTC	EuUCBA	red→colorless		0.118 μM	[168]
CTC		red→colorless		0.228 μM	
MTC		red→colorless		0.102 μM	
MOC		red→colorless		0.138 μM	
TC		red→colorless		0.206 μM	
DOXY		red→dark		0.078 μM	
DCNA	Eu^3+^@Zn-MOF-NS	red→pink		0.17 μM	[169]
4-NA	Eu^3+^@Zn-MOF	red→dark		6.01 μM	[170]
Rotenone	Eu@Zn-MOF	red→dark		2.31 × 10^−7^ mol/L	[171]
Carbaryl	Eu^3+^@MOF-253	red→yellow	0.2–200 µg/L	0.14 μg/L	[172]
TBZ	Tb^3+^@MOF	green→blue	0–80 μM	0.271 μM	[173]
H2S	Tb^3+^@MOF	blue→green	10–600 µM	1.20 μM	[174]
H2O	Tb^3+^@pCDs/MOF	red→green	0–30%	0.28%	[175]
Pesticides	AuNCs@ZIF-8	blue→colorless	0.75 μg/L–100 mg/L	0.4 μg/L	[158]
PA	RGH-Eu(BTC)	orange→yellow	0–100 μM	0.45 μM	[176]
ALP	SQDs@ZIF-8	blue→colorless	0.15–50 U/L	0.044 U/L	[177]
GLP	N-CDs@MOF	blue→colorless	0.01–6.67 mg/L	9.06 µg/L	[178]
Ammonia	ZnQ@Zn-BTC	pink→green	0.1–2 mg/L	0.27 mg/L	[179]
Kanamycin	ZIF8@TPE/Aptamer	green→blue	10–103 ng/mL	7.3 ng/mL	[180]
OTC	NH2-BDC@FMIL-53(Al)-3	blue→green	0.3–4.0 μM	0.18 μM	[181]

Abbreviations: 1-N, 1-naphthol; OTC, oxytetracycline; CTC, chlortetracycline; MTC, methylenetetracycline; MOC, minocycline; TC, tetracycline; DOXY, doxycycline; TBZ, thiabendazole; 4-NA, 4-nitroaniline; BPA, bisphenol A; QD, quantum dot; CQD, carbon quantum dots; PAT, mycotoxin patulin; DCNA, 2,6-dichloro-4-nitroaniline; RGH, rhodamine derivative; PA, picric acid; GLP, glyphosate; OTC, oxytetracycline.

**Table 2:** Performances of MOF-based fluorescent chemosensors for the detection of inorganic ions.

Targets	MOF	Color Change	Linear Range	LOD	Ref.
Fe^3+^	CD@Eu-MOF	purple→red	1–200 µM	0.91 µM	[170]
	Eu^3+^/CDs@MOF	red→colorless	0–6 μM	0.034 μM	[182]
	Tb^3+^@UiO6(COOH)	green→blue	0–200 μM	0.23 μM	[183]
	SRB@UiO-66	red→pink	0.1–0.9 mM	3.693 μM	[184]
	Dye@bio-MOF-1	green→dark	10^−5^–10^−2^ M		[185]
Cu^2+^	ZnS QDs@ZIF-8	yellow→blue	0.05–5 μM	16 nM	[186]
	BPEI-CQDs/ZIF-8	blue→colorless	2–1000 nM	80 pM	[187]
	ZTMs@FITC	red→green	0.1–5 μM	5.61 nM	[188]
Hg^2+^	Eu^3+^/CDs@MOF-253	blue→red	0.065–150 μM	13 μg/L	[82]
	CDs@Eu-MOFs	blue→red	0–300 μM	0.12 nM	[189]
Ag^+^	Eu^3+^@MIL-121	colorless→red	0–100 μM	0.1 μM	[190]
Pb^2+^	CDs/QDs@ZIF-8	red→blue	0.04–60 μM		[79]
PO_4_^3−^	CDs/QDs@ZIF-8	blue→red	0.25–50 μM	9.42 nM	[79]

**Table 3:** Comparison of the performance of the techniques/strategies developed to detect Fe^3+^ with the platform using fluorescence, presented along with their colorimetric method, limit of detection (LOD), and the linear sensing range.

Sl. No	Strategy/Approach Adopted	Nanomaterial Used	Colorimetric	LOD	Linear Range	Ref.
1	Electrochemical method	Cu-MOF		14.5 fM		[191]
2	Electrochemical method	Tb-MOF		4.84 μM		[192]
3	Electrochemical method	Iodide-enhanced Cu-MOF		200 nM		[193]
4	SPCE platform	C-dots modified screen printed carbon electrode (SPCE)		0.44 ppm	0.5–25 ppm	[194]
5	Spectrophotometry	Casein-capped gold nanoparticles (AuNPs)		450 nM	0.1–0.9 μM	[195]
6	Surface-enhanced Raman spectroscopy (SERS)	Phenanthroline probe		0.001 ppm	0.01–0.001 ppm	[196]
7	Voltammetry (cyclic voltammetry and differential pulse voltammetry)	Quercetin		3.6 nM	17.9–716.0 nM	[197]
8	Electrochemical method	N, S doped GQD		0.23 nM	1–100 nM	[198]
9	Ion chromatography	Ionic liquids		0.09 ppm	1–100 ppm	[199]
10	Interferometric optical microfiber method	Ditrogen- and sulfur-codoped CDs		0.77 μg/L	0–300 μg/L	[200]
11	Surface plasmon resonance optical detection	CTAB/hydroxylated graphene quantum dots		0.1 ppm		[201]
12	Fluorescence	CD@Eu-MOF	blue→pink	0.91 µM	1–200 µM	[202]
13	Fluorescence	Acf@bioMOF	yellow→colorless	1.33 μM	0–370 μM	[139]
14	Fluorescence	EY@Zr-MOF	blue→yellow	0.1 μM	0–1 mM	[137]

**Table 4:** Summary table of the comparison of the performance of the recent techniques/strategies developed for biosensing with fluorescence spectroscopy, presented along with their advantages and disadvantages.

Sl. No.	Strategy/Approach Adopted	Methodology in Brief	Advantages	Disadvantages	Ref.
1	Electrochemical method	Studying the interconversion of chemical and electrical energy and the related phenomena and laws in the process of conversion.	Fast response time High sensitivity Selectivity The possibility to achieve real-time measurements.	Short life span Limited temperature range	[203,204]
2	Spectrophotometry	Qualitative and quantitative analysis of a measured substance, by measuring the absorbance of light at a specific wavelength or in a certain wavelength range.	Simple instrumentation, easy and fast operation	Relatively inaccurate	[205,206,207]
3	Surface plasmon coupled emission (SPCE)	Prism-coupling technique, where metallic thin film is coupled to the prism and the emission is monitored via a filter, a polarizer, an optic fiber, and a detector.	High surface EM field intensity on account of generation of SPPs Low background noise High signal collection	Multiple uses of the same substrate are not possible High Ohmic losses High surface-induced quenching effects observed	[208,209]
4	Surface-enhanced Raman spectroscopy (SERS)	Determination of samples adsorbed on colloidal metal particles such as silver, gold, or copper, or on the rough surfaces of these metal sheets.	High detection sensitivity Fast analysis speed Low concentration and non-destructive sample required	Difficult to control base line relinearity and stability	[210,211,212]
5	Ion chromatography	It is a liquid chromatographic method for the analysis of anions and cations and belongs to the category of high-performance liquid chromatography (HPLC).	High capacity and stability of ion chromatography separation columns	Poor qualitative ability	[213,214,215]
6	Fluorescence spectroscopy	The emission from the fluorescent molecules present in the cuvette is captured using a detector placed at 90° to the light source.	High sensitivity Highly versatile method Portable visual fluorescence sensing compared to other method	High background noise Low spectral resolution	[215,216,217,218]

## Data Availability

Not applicable.

## References

[1] Lin X., Chen Q., Liu W., Li H., Lin J.-M. (2014). A portable microchip for ultrasensitive and high-throughput assay of thrombin by rolling circle amplification and hemin/G-quadruplex system. Biosens. Bioelectron..

[2] Chin C.D., Laksanasopin T., Cheung Y.K., Steinmiller D., Linder V., Parsa H., Wang J., Moore H., Rouse R., Umviligihozo G. (2011). Microfluidics-based diagnostics of infectious diseases in the developing world. Nat. Med..

[3] Lauwers D., Hutado A.G., Tanevska V., Moens L., Bersani D., Vandenabeele P. (2014). Characterisation of a portable Raman spectrometer for in situ analysis of art objects. Spectrochim. Acta Part A Mol. Biomol. Spectrosc..

[4] Lee W.G., Kim Y.-G., Chung B.G., Demirci U., Khademhosseini A. (2010). Nano/Microfluidics for diagnosis of infectious diseases in developing countries. Adv. Drug Deliv. Rev..

[5] Xiang Y., Lu Y. (2012). Portable and Quantitative Detection of Protein Biomarkers and Small Molecular Toxins Using Antibodies and Ubiquitous Personal Glucose Meters. Anal. Chem..

[6] Rathnakumar S., Bhaskar S., Rai A., Saikumar D.V.V., Kambhampati N.S.V., Sivaramakrishnan V., Ramamurthy S.S. (2021). Plasmon-Coupled Silver Nanoparticles for Mobile Phone-Based Attomolar Sensing of Mercury Ions. ACS Appl. Nano Mater..

[7] Zheng P., Raj P., Wu L., Szabo M., Hanson W.A., Mizutani T., Barman I. (2022). Leveraging Nanomechanical Perturbations in Raman Spectro-Immunoassays to Design a Versatile Serum Biomarker Detection Platform. Small.

[8] Imran M., Ahmed S., Abdullah A.Z., Hakami J., Chaudhary A.A., Rudayni H.A., Khan S., Khan A., Basher N.S. (2022). Nanostructured material-based optical and electrochemical detection of amoxicillin antibiotic. Luminescence.

[9] Ahmed S., Ansari A., Haidyrah A.S., Chaudhary A.A., Imran M., Khan A. (2022). Hierarchical Molecularly Imprinted Inverse Opal-Based Platforms for Highly Selective and Sensitive Determination of Histamine. ACS Appl. Polym. Mater..

[10] Fang L., Liao X., Jia B., Shi L., Kang L., Zhou L., Kong W. (2020). Recent progress in immunosensors for pesticides. Biosens. Bioelectron..

[11] Zhang Y., Wu Q., Sun M., Zhang J., Mo S., Wang J., Wei X., Bai J. (2018). Magnetic-assisted aptamer-based fluorescent assay for allergen detection in food matrix. Sens. Actuators B Chem..

[12] Luo L., Song Y., Zhu C., Fu S., Shi Q., Sun Y.-M., Jia B., Du D., Xu Z.-L., Lin Y. (2017). Fluorescent silicon nanoparticles-based ratiometric fluorescence immunoassay for sensitive detection of ethyl carbamate in red wine. Sens. Actuators B Chem..

[13] Shamsipur M., Molaabasi F., Hosseinkhani S., Rahmati F. (2016). Detection of Early Stage Apoptotic Cells Based on Label-Free Cytochrome c Assay Using Bioconjugated Metal Nanoclusters as Fluorescent Probes. Anal. Chem..

[14] Bik E., Mateuszuk L., Stojak M., Chlopicki S., Baranska M., Majzner K. (2020). Menadione-induced endothelial inflammation detected by Raman spectroscopy. Biochim. Et Biophys. Acta (BBA)-Mol. Cell Res..

[15] Mikac L., Kovačević E., Ukić Š., Raić M., Jurkin T., Marić I., Gotić M., Ivanda M. (2021). Detection of multi-class pesticide residues with surface-enhanced Raman spectroscopy. Spectrochim. Acta Part A Mol. Biomol. Spectrosc..

[16] Boardman A.K., Wong W.S., Premasiri W.R., Ziegler L.D., Lee J.C., Miljkovic M., Klapperich C.M., Sharon A., Sauer-Budge A.F. (2016). Rapid Detection of Bacteria from Blood with Surface-Enhanced Raman Spectroscopy. Anal. Chem..

[17] Ding Y., Zhang X., Yin H., Meng Q., Zhao Y., Liu L., Wu Z., Xu H. (2017). Quantitative and Sensitive Detection of Chloramphenicol by Surface-Enhanced Raman Scattering. Sensors.

[18] Liu Y., Chen Y., Zhang Y., Kou Q., Zhang Y., Wang Y., Chen L., Sun Y., Zhang H., MeeJung Y. (2018). Detection and Identification of Estrogen Based on Surface-Enhanced Resonance Raman Scattering (SERRS). Molecules.

[19] Lv M., Liu Y., Geng J., Kou X., Xin Z., Yang D. (2018). Engineering nanomaterials-based biosensors for food safety detection. Biosens. Bioelectron..

[20] Yarur F., Macairan J.-R., Naccache R. (2019). Ratiometric detection of heavy metal ions using fluorescent carbon dots. Environ. Sci. Nano.

[21] Ze Y., Yi C., Jing Z., Yong Z. (2021). Fluorescent sensor arrays for metal ions detection: A review. Measurement.

[22] Guo Y., Zhang L., Zhang S., Yang Y., Chen X., Zhang M. (2014). Fluorescent carbon nanoparticles for the fluorescent detection of metal ions. Biosens. Bioelectron..

[23] Sharma P., Mehata M.S. (2020). Rapid sensing of lead metal ions in an aqueous medium by MoS2 quantum dots fluorescence turn-off. Mater. Res. Bull..

[24] Singh J., Kaur S., Lee J., Mehta A., Kumar S., Kim K.-H., Basu S., Rawat M. (2020). Highly fluorescent carbon dots derived from Mangifera indica leaves for selective detection of metal ions. Sci. Total Environ..

[25] Xu Q., Li J., Gong X. (2022). Dual-emission carbon dots for sensitive fluorescence detection of metal ions and ethanol in water. Anal. Methods.

[26] Ma S., Ji R., Wang X., Yu C., Yu Y., Yang X. (2018). Fluorescence detection of boscalid pesticide residues in grape juice. Optik.

[27] Watthaisong P., Kamutira P., Kesornpun C., Pongsupasa V., Phonbuppha J., Tinikul R., Maenpuen S., Wongnate T., Nishihara R., Ohmiya Y. (2022). Luciferin Synthesis and Pesticide Detection by Luminescence Enzymatic Cascades. Angew. Chem. Int. Ed..

[28] Ashrafi Tafreshi F., Fatahi Z., Ghasemi S.F., Taherian A., Esfandiari N. (2020). Ultrasensitive fluorescent detection of pesticides in real sample by using green carbon dots. PLoS ONE.

[29] Huasheng M., Mamoun C., Xiangyang W., Lili X., Lijun Y., Jianying H. (2022). Fluorescent Detection of Organophosphorus Pesticides Using Carbon Dots Derived from Broccoli. Arab. J. Sci. Eng..

[30] Lin B., Yan Y., Guo M., Cao Y., Yu Y., Zhang T., Huang Y., Wu D. (2017). Modification-free carbon dots as turn-on fluorescence probe for detection of organophosphorus pesticides. Food Chem..

[31] Nsibande S.A., Forbes P.B.C. (2016). Fluorescence detection of pesticides using quantum dot materials—A review. Anal. Chim. Acta.

[32] Wang J., Zhang J., Wang J., Fang G., Liu J., Wang S. (2020). Fluorescent peptide probes for organophosphorus pesticides detection. J. Hazard. Mater..

[33] Yang J., Chen S.-W., Zhang B., Tu Q., Wang J., Yuan M.-S. (2022). Non-biological fluorescent chemosensors for pesticides detection. Talanta.

[34] Haibo L., Yumin W., Li Z., Yujuan C., Manli G., Ying Y., Bixia L. (2022). Construction of integrated and portable fluorescence sensor and the application for visual detection in situ. Sens. Actuators B Chem..

[35] Deb A., Nalkar G.R., Chowdhury D. (2023). Biogenic carbon dot-based fluorescence-mediated immunosensor for the detection of disease biomarker. Anal. Chim. Acta.

[36] Li Y., Jia D., Ren W., Shi F., Liu C. (2019). A Versatile Photoinduced Electron Transfer-Based Upconversion Fluorescent Biosensing Platform for the Detection of Disease Biomarkers and Nerve Agent. Adv. Funct. Mater..

[37] Sun Z.-H., Zhang X.-X., Xu D., Liu J., Yu R.-J., Jing C., Han H.-X., Ma W. (2020). Silver-amplified fluorescence immunoassay via aggregation-induced emission for detection of disease biomarker. Talanta.

[38] Yao B., Giel M.-C., Hong Y. (2021). Detection of kidney disease biomarkers based on fluorescence technology. Mater. Chem. Front..

[39] Yao C., Liu Q., Zhao N., Liu J.-M., Fang G., Wang S. (2021). Ratiometric determination of Cr(VI) based on a dual-emission fluorescent nanoprobe using carbon quantum dots and a smartphone app. Microchim. Acta.

[40] Kirchon A., Feng L., Drake H.F., Joseph E.A., Zhou H.-C. (2018). From fundamentals to applications: A toolbox for robust and multifunctional MOF materials. Chem. Soc. Rev..

[41] Zhou H.-C., Long J.R., Yaghi O.M. (2012). Introduction to Metal–Organic Frameworks. Chem. Rev..

[42] Cheng X.-B., Zhao C.-Z., Yao Y.-X., Liu H., Zhang Q. (2019). Recent Advances in Energy Chemistry between Solid-State Electrolyte and Safe Lithium-Metal Anodes. Chem.

[43] Yan F., Wang X., Wang Y., Yi C., Xu M., Xu J. (2022). Sensing performance and mechanism of carbon dots encapsulated into metal–organic frameworks. Microchim. Acta.

[44] Yin H.-Q., Yin X.-B. (2020). Metal-Organic Frameworks with Multiple Luminescence Emissions: Designs and Applications. Acc. Chem. Res..

[45] Harine G., Ajay V.M., Rajkumar P., Bhaskar R.S. (2023). Metal-Organic Framework Derived Carbon-based Electrocatalysis for Hydrogen Evolution Reactions: A Review. Mater. Today Sustain..

[46] Kundu S., Swaroop A.K., Selvaraj J. (2023). Metal-Organic Framework in Pharmaceutical Drug Delivery. Curr. Top. Med. Chem..

[47] Li B., Dong J.-P., Zhou Z., Wang R., Wang L.-Y., Zang S.-Q. (2021). Robust lanthanide metal–organic frameworks with “all-in-one” multifunction: Efficient gas adsorption and separation, tunable light emission and luminescence sensing. J. Mater. Chem. C.

[48] Kaur H., Sundriyal S., Pachauri V., Ingebrandt S., Kim K.-H., Sharma A.L., Deep A. (2019). Luminescent metal-organic frameworks and their composites: Potential future materials for organic light emitting displays. Coord. Chem. Rev..

[49] Lustig W.P., Li J. (2018). Luminescent metal–organic frameworks and coordination polymers as alternative phosphors for energy efficient lighting devices. Coord. Chem. Rev..

[50] Dong J., Zhao D., Lu Y., Sun W.-Y. (2019). Photoluminescent metal–organic frameworks and their application for sensing biomolecules. J. Mater. Chem. A.

[51] Zhang Y., Yuan S., Day G., Wang X., Yang X., Zhou H.-C. (2018). Luminescent sensors based on metal-organic frameworks. Coord. Chem. Rev..

[52] Hardian R., Dissegna S., Ullrich A., Llewellyn P.L., Coulet M.-V., Fischer R.A. (2021). Tuning the Properties of MOF-808 via Defect Engineering and Metal Nanoparticle Encapsulation. Chem. Eur. J..

[53] Liu X., Hu H., Liu Y., Huang Z., Lu Y., Zhou X., Wang J. (2020). Experimental investigation on fluorescence polarization properties of isomerical MOF⊃RhB crystals. J. Solid State Chem..

[54] Qiu L., Yu C., Wang X., Xie Y., Kirillov A.M., Huang W., Li J., Gao P., Wu T., Gu X. (2019). Tuning the Solid-State White Light Emission of Postsynthetic Lanthanide-Encapsulated Double-Layer MOFs for Three-Color Luminescent Thermometry Applications. Inorg. Chem..

[55] Li G., Zhao S., Zhang Y., Tang Z. (2018). Metal–Organic Frameworks Encapsulating Active Nanoparticles as Emerging Composites for Catalysis: Recent Progress and Perspectives. Adv. Mater..

[56] Mollick S., Mandal T.N., Jana A., Fajal S., Desai A.V., Ghosh S.K. (2019). Ultrastable Luminescent Hybrid Bromide [email protected] Nanocomposites for the Degradation of Organic Pollutants in Water. ACS Appl. Energy Mater..

[57] Xu Z., Zhang J., Pan T., Li H., Huo F., Zheng B., Zhang W. (2020). Encapsulation of Hydrophobic Guests within Metal–Organic Framework Capsules for Regulating Host–Guest Interaction. Chem. Mater..

[58] Peng M., Kaczmarek A.M., Van Hecke K. (2022). Ratiometric Thermometers Based on Rhodamine B and Fluorescein Dye-Incorporated (Nano) Cyclodextrin Metal–Organic Frameworks. ACS Appl. Mater. Interfaces.

[59] Jiang X., Fan R., Zhang J., Fang X., Sun T., Zhu K., Zhou X., Xu Y., Yang Y. (2022). Sequentially epitaxial growth multi-guest encapsulation strategy in MOF-on-MOF platform: Biogenic amine detection and systematic white light adjustment. Chem. Eng. J..

[60] Xia Q.Q., Wang X.H., Yu J.L., Xue Z.Y., Chai J., Wu M.X., Liu X.M. (2022). Tunable fluorescence emission based on multi-layered MOF-on-MOF. Dalton Trans..

[61] Xu D.-D., Dong W.-W., Li M.-K., Han H.-M., Zhao J., Li D.-S., Zhang Q. (2022). Encapsulating Organic Dyes in Metal–Organic Frameworks for Color-Tunable and High-Efficiency White-Light-Emitting Properties. Inorg. Chem..

[62] Yi K., Zhang X., Zhang L. (2020). Eu^3+^@metal-organic frameworks encapsulating carbon dots as ratiometric fluorescent probes for rapid recognition of anthrax spore biomarker. Sci. Total Environ..

[63] Yin H.-Q., Yang J.-C., Yin X.-B. (2017). Ratiometric Fluorescence Sensing and Real-Time Detection of Water in Organic Solvents with One-Pot Synthesis of [email protected](Al)–NH_2_. Anal. Chem..

[64] Ma Y., Zhang X., Bai J., Huang K., Ren L. (2019). Facile, controllable tune of blue shift or red shift of the fluorescence emission of solid-state carbon dots. Chem. Eng. J..

[65] Rodrigues M.O., Paz F.A.A., Freire R.O., de Sá G.F., Galembeck A., Montenegro M.C.B.S.M., Araújo A.N., Alves S. (2009). Modeling, Structural, and Spectroscopic Studies of Lanthanide-Organic Frameworks. J. Phys. Chem. B.

[66] Yin Z., Wan S., Yang J., Kurmoo M., Zeng M.-H. (2017). Recent advances in post-synthetic modification of metal–organic frameworks: New types and tandem reactions. Coord. Chem. Rev..

[67] Wang F., Zhang F., Zhao Z., Sun Z., Pu Y., Wang Y., Wang X. (2021). Multifunctional MOF-based probes for efficient detection and discrimination of Pb^2+^, Fe^3+^ and Cr_2_O_7_^2−^/CrO_4_^2−^. Dalton Trans..

[68] Liang Y.-Y., Luo L.-J., Li Y., Ling B.-K., Chen B.-W., Wang X.-W., Luan T.-G. (2018). A Luminescent Probe for Highly Selective Cu^2+^ Sensing Using a Lanthanide-Doped Metal Organic Framework with Large Pores. Eur. J. Inorg. Chem..

[69] Xiao J., Song L., Liu M., Wang X., Liu Z. (2020). Intriguing pH-modulated Luminescence Chameleon System based on Postsynthetic Modified Dual-emitting Eu^3+^@Mn-MOF and Its Application for Histidine Chemosensor. Inorg. Chem..

[70] Luo J., Liu B.-S., Zhang X.-R., Liu R.-T. (2019). A Eu^3+^ post-functionalized metal-organic framework as fluorescent probe for highly selective sensing of Cu^2+^ in aqueous media. J. Mol. Struct..

[71] Xu X.-Y., Yan B. (2014). Eu(III)-Functionalized MIL-124 as Fluorescent Probe for Highly Selectively Sensing Ions and Organic Small Molecules Especially for Fe(III) and Fe(II). ACS Appl. Mater. Interfaces.

[72] Ji G., Gao X., Zheng T., Guan W., Liu H., Liu Z. (2018). Postsynthetic Metalation Metal–Organic Framework as a Fluorescent Probe for the Ultrasensitive and Reversible Detection of PO_4_^3–^ Ions. Inorg. Chem..

[73] Wu J.-X., Yan B. (2018). Luminescent Hybrid Tb^3+^ Functionalized Metal–Organic Frameworks Act as Food Preservative Sensor and Water Scavenger for NO^2–^. Ind. Eng. Chem. Res..

[74] Wang Y., Yang H., Cheng G., Wu Y., Lin S. (2017). A new Tb(iii)-functionalized layer-like Cd MOF as luminescent probe for high-selectively sensing of Cr^3+^. CrystEngComm.

[75] Yu W.W., Qu L., Guo W., Peng X. (2003). Experimental Determination of the Extinction Coefficient of CdTe, CdSe, and CdS Nanocrystals. Chem. Mater..

[76] Bajorowicz B., Kobylański M.P., Gołąbiewska A., Nadolna J., Zaleska-Medynska A., Malankowska A. (2018). Quantum dot-decorated semiconductor micro- and nanoparticles: A review of their synthesis, characterization and application in photocatalysis. Adv. Colloid Interface Sci..

[77] Asadi F., Azizi S.N., Chaichi M.J. (2019). Green synthesis of fluorescent PEG-ZnS QDs encapsulated into Co-MOFs as an effective sensor for ultrasensitive detection of copper ions in tap water. Mater. Sci. Eng. C.

[78] Ma Y., Xu G., Wei F., Cen Y., Ma Y., Song Y., Xu X., Shi M., Muhammad S., Hu Q. (2017). A dual-emissive fluorescent sensor fabricated by encapsulating quantum dots and carbon dots into metal–organic frameworks for the ratiometric detection of Cu^2+^ in tap water. J. Mater. Chem. C.

[79] Yi K., Zhang L. (2020). Embedding dual fluoroprobe in metal-organic frameworks for continuous visual recognition of Pb^2+^ and PO_4_^3-^ via fluorescence 'turn-off-on' response: Agar test paper and fingerprint. J. Hazard. Mater..

[80] Ma C., Li P., Xia L., Qu F., Kong R.-M., Song Z.-L. (2021). A novel ratiometric fluorescence nanoprobe for sensitive determination of uric acid based on CD@ZIF-CuNC nanocomposites. Microchim. Acta.

[81] Hao J., Liu F., Liu N., Zeng M., Song Y., Wang L. (2017). Ratiometric fluorescent detection of Cu^2+^ with carbon dots chelated Eu-based metal-organic frameworks. Sens. Actuators B Chem..

[82] Xu X.-Y., Yan B. (2016). Fabrication and application of a ratiometric and colorimetric fluorescent probe for Hg^2+^ based on dual-emissive metal–organic framework hybrids with carbon dots and Eu^3+^. J. Mater. Chem. C.

[83] Chen R., Zhang J., Chelora J., Xiong Y., Kershaw S.V., Li K.F., Lo P.-K., Cheah K.W., Rogach A.L., Zapien J.A. (2017). Ruthenium(II) Complex Incorporated UiO-67 Metal–Organic Framework Nanoparticles for Enhanced Two-Photon Fluorescence Imaging and Photodynamic Cancer Therapy. ACS Appl. Mater. Interfaces.

[84] Sun C.-Y., Wang X.-L., Zhang X., Qin C., Li P., Su Z.-M., Zhu D.-X., Shan G.-G., Shao K.-Z., Wu H. (2013). Efficient and tunable white-light emission of metal-organic frameworks by iridium-complex encapsulation. Nat. Commun..

[85] Zhao H., Ni J., Zhang J.-J., Liu S.-Q., Sun Y.-J., Zhou H., Li Y.-Q., Duan C.-Y. (2018). A trichromatic MOF composite for multidimensional ratiometric luminescent sensing. Chem. Sci..

[86] Zhu S., Wang S., Xia M., Wang B., Huang Y., Zhang D., Zhang X., Wang G. (2019). Intracellular Imaging of Glutathione with MnO2 Nanosheet@Ru(bpy)_3_^2+^-UiO-66 Nanocomposites. ACS Appl. Mater. Interfaces.

[87] Fu Y., Finney N.S. (2018). Small-molecule fluorescent probes and their design. RSC Adv..

[88] Traven V.F., Cheptsov D.A. (2020). Sensory effects of fluorescent organic dyes. Russ. Chem. Rev..

[89] Xu X.-Y., Yan B., Lian X. (2018). Wearable glove sensor for non-invasive organophosphorus pesticide detection based on a double-signal fluorescence strategy. Nanoscale.

[90] Liu N., Hao J., Chen L., Song Y., Wang L. (2019). Ratiometric fluorescent detection of Cu^2+^ based on dual-emission ZIF-8@rhodamine-B nanocomposites. Luminescence.

[91] Feng D.Y., Zhang T., Zhong T.Y., Zhang C., Tian Y.Y., Wang G. (2021). Coumarin-embedded MOF UiO-66 as a selective and sensitive fluorescent sensor for the recognition and detection of Fe^3+^ ions. J. Mater. Chem. C.

[92] Li H., Fu F., Yang W., Ding L., Dong J., Yang Y., Wang F., Pan Q. (2019). A simple fluorescent probe for fast and sensitive detection of inorganic phosphate based on [email protected] composite. Sens. Actuators B Chem..

[93] Huang C.J., Ye Y.X., Zhao L.W., Li Y.S., Gu J.L. (2019). One-Pot Trapping Luminescent Rhodamine 110 into the Cage of MOF-801 for Nitrite Detection in Aqueous Solution. J. Inorg. Organomet. Polym. Mater..

[94] Jin R. (2009). Quantum sized, thiolate-protected gold nanoclusters. Nanoscale.

[95] Burrows P.E., Sapochak L.S., McCarty D.M., Forrest S.R., Thompson M.E. (1994). Metal ion dependent luminescence effects in metal tris-quinolate organic heterojunction light emitting devices. Appl. Phys. Lett..

[96] Goswami N., Lin F., Liu Y., Leong D.T., Xie J. (2016). Highly Luminescent Thiolated Gold Nanoclusters Impregnated in Nanogel. Chem. Mater..

[97] Luo Z., Yuan X., Yu Y., Zhang Q., Leong D.T., Lee J.Y., Xie J. (2012). From Aggregation-Induced Emission of Au(I)–Thiolate Complexes to Ultrabright Au(0)@Au(I)–Thiolate Core–Shell Nanoclusters. J. Am. Chem. Soc..

[98] Han B., Hu X., Yu M., Peng T., Li Y., He G. (2018). One-pot synthesis of enhanced fluorescent copper nanoclusters encapsulated in metal–organic frameworks. RSC Adv..

[99] Pirot S.M., Omer K.M. (2022). Designing of robust and sensitive assay via encapsulation of highly emissive and stable blue copper nanocluster into zeolitic imidazole framework (ZIF-8) with quantitative detection of tetracycline. J. Anal. Sci. Technol..

[100] Chen H., Chang Y., Wei R., Zhang P. (2022). Gold nanoclusters encapsulated into zinc-glutamate metal organic frameworks for efficient detection of H_2_O_2_. Anal. Methods.

[101] Liu P., Hao R., Sun W., Lin Z., Jing T. (2022). One-pot synthesis of copper nanocluster/Tb-MOF composites for the ratiometric fluorescence detection of Cu^2+^. Luminescence.

[102] Jalili R., Irani-Nezhad M.H., Khataee A., Joo S.W. (2021). A ratiometric fluorescent probe based on carbon dots and gold nanocluster encapsulated metal–organic framework for detection of cephalexin residues in milk. Spectrochim. Acta Part A Mol. Biomol. Spectrosc..

[103] Hu J., Cui X., Gong Y., Xu X., Gao B., Wen T., Lu T.J., Xu F. (2016). Portable microfluidic and smartphone-based devices for monitoring of cardiovascular diseases at the point of care. Biotechnol. Adv..

[104] Jongs N., Jagesar R., Koning I., Ruhe H., Van Haren N., Vorstman J., Kas M. (2018). Passive behavioural monitoring in neuropsychiatric disorders using smartphone technology. Eur. Neuropsychopharmacol..

[105] Majumder S., Deen M.J. (2019). Smartphone Sensors for Health Monitoring and Diagnosis. Sensors.

[106] Moses J.C., Adibi S., Shariful Islam S.M., Wickramasinghe N., Nguyen L. (2021). Application of Smartphone Technologies in Disease Monitoring: A Systematic Review. Healthcare.

[107] Andrachuk M., Marschke M., Hings C., Armitage D. (2019). Smartphone technologies supporting community-based environmental monitoring and implementation: A systematic scoping review. Biol. Conserv..

[108] Chen Z.s., Liu T., Dong J.f., Chen G., Li Z., Zhou J.l., Chen Z. (2023). Sustainable Application for Agriculture Using Biochar-Based Slow-Release Fertilizers: A Review. ACS Sustain. Chem. Eng..

[109] Ramar R., Malaichamy I. (2021). Simple smartphone merged rapid colorimetric platform for the environmental monitoring of toxic sulfide ions by cysteine functionalized silver nanoparticles. Microchem. J..

[110] Wang Y., Tan R., Xing G., Wang J., Tan X., Liu X. (2016). Energy-Efficient Aquatic Environment Monitoring Using Smartphone-Based Robots. ACM Trans. Sens. Netw..

[111] Deng C.C., Xu Z.Y., Sun Z., Xie J.H., Luo H.Q., Li N.B. (2022). One-step synthesis of aldehyde-functionalized dual-emissive carbon dots for ratiometric fluorescence detection of bisulfite in food samples. Food Chem..

[112] Rateni G., Dario P., Cavallo F. (2017). Smartphone-Based Food Diagnostic Technologies: A Review. Sensors.

[113] Zhang J., Huang H., Song G., Huang K., Luo Y., Liu Q., He X., Cheng N. (2022). Intelligent biosensing strategies for rapid detection in food safety: A review. Biosens. Bioelectron..

[114] Bhaskar S., Ramamurthy S.S. (2019). Mobile Phone-Based Picomolar Detection of Tannic Acid on Nd2O3 Nanorod–Metal Thin-Film Interfaces. ACS Appl. Energy Mater..

[115] Aayush R., Seemesh B., Kalathur Mohan G., Sai Sathish R. (2021). Engineering of coherent plasmon resonances from silver soret colloids, graphene oxide and Nd_2_O_3_ nanohybrid architectures studied in mobile phone-based surface plasmon-coupled emission platform. Mater. Lett..

[116] Rai A., Bhaskar S., Reddy N., Ramamurthy S.S. (2021). Cellphone-Aided Attomolar Zinc Ion Detection Using Silkworm Protein-Based Nanointerface Engineering in a Plasmon-Coupled Dequenched Emission Platform. ACS Sustain. Chem. Eng..

[117] Seemesh B., Dipin T., Sai Sathish R., Chandramouli S. (2022). Metal–Dielectric Interfacial Engineering with Mesoporous Nano-Carbon Florets for 1000-Fold Fluorescence Enhancements: Smartphone-Enabled Visual Detection of Perindopril Erbumine at a Single-molecular Level. ACS Sustain. Chem. Eng..

[118] Bhaskar S., Ramamurthy S.S. (2021). Synergistic coupling of titanium carbonitride nanocubes and graphene oxide for 800-fold fluorescence enhancements on smartphone based surface plasmon-coupled emission platform. Mater. Lett..

[119] Xiaoting Z., Ying L., Lei Z. (2021). Developed ratiometric fluorescent probe as a logic platform for potential diagnosis of thyroid disease and diabetes and fluorescent ink. Microchem. J..

[120] Kou X., Tong L., Shen Y., Zhu W., Yin L., Huang S., Zhu F., Chen G., Ouyang G. (2020). Smartphone-assisted robust enzymes@MOFs-based paper biosensor for point-of-care detection. Biosens. Bioelectron..

[121] Li Z., Xi Y., Zhao A., Jiang J., Li B., Yang X., He J., Li F. (2021). Cobalt-imidazole metal-organic framework loaded with luminol for paper-based chemiluminescence detection of catechol with use of a smartphone. Anal. Bioanal. Chem..

[122] Yang L., Liu Y., Chen L., Guo L., Lei Y., Wang L. (2021). Stable dual-emissive fluorescin@UiO-67 metal-organic frameworks for visual and ratiometric sensing of Al^3+^ and ascorbic acid. Spectrochim. Acta Part A Mol. Biomol. Spectrosc..

[123] Wang B.H., Yan B. (2019). A dye@MOF crystalline probe serving as a platform for ratiometric sensing of trichloroacetic acid (TCA), a carcinogen metabolite in human urine. CrystEngComm.

[124] Fu X., Lv R., Su J., Li H., Yang B., Gu W., Liu X. (2018). A dual-emission nano-rod MOF equipped with carbon dots for visual detection of doxycycline and sensitive sensing of MnO^4−^. RSC Adv..

[125] Wang L.-B., Wang J.-J., Yue E.-L., Tang L., Wang X., Hou X.-Y., Zhang Y., Ren Y.-X., Chen X.-L. (2021). Highly selective detecting Aspartic acid, detecting Ornidazole and information encryption and decryption supported by a heterometallic anionic Cd (II)-K (I)-MOF. Spectrochim. Acta Part A Mol. Biomol. Spectrosc..

[126] Xiaomeng Z., Xinjie W., Li S. (2022). Ratiometric fluorescence and visual sensing of ATP based on gold nanocluster-encapsulated metal-organic framework with a smartphone. Chin. Chem. Lett..

[127] Xiang-Juan K., Jing-Xuan T., Yan-Zhao F., Tao-Li C., Rui Y., Jia-Yu H., Zi-Yan Z., Qiang X. (2021). Terbium metal-organic framework/bovine serum albumin capped gold nanoclusters-based dual-emission reverse change ratio fluorescence nanoplatform for fluorimetric and colorimetric sensing of heparin and chondroitin sulfate. Sens. Actuators B Chem..

[128] Zhang Y., Zhang Y., Li L., Chen J., Li P., Huang W. (2020). One-step in situ growth of high-density POMOFs films on carbon cloth for the electrochemical detection of bromate. J. Electroanal. Chem..

[129] Wang C., Tian L., Zhu W., Wang S.Q., Wang P., Liang Y., Zhang W.L., Zhao H.W., Li G.T. (2017). Dye@bio-MOF-1 Composite as a Dual-Emitting Platform for Enhanced Detection of a Wide Range of Explosive Molecules. Acs Appl. Mater. Interfaces.

[130] Wang H.J., Sha Z.J. (2011). Preparation of copper net-supported metal-organic framework-5 membranes for solid-state lasers. Sci. China-Chem..

[131] Gao N., Huang J., Wang L.Y., Feng J.Y., Huang P.C., Wu F.Y. (2018). Ratiometric fluorescence detection of phosphate in human serum with a metal-organic frameworks-based nanocomposite and its immobilized agarose hydrogels. Appl. Surf. Sci..

[132] Wei D., Li M., Wang Y., Zhu N., Hu X., Zhao B., Zhang Z., Yin D. (2023). Encapsulating gold nanoclusters into metal–organic frameworks to boost luminescence for sensitive detection of copper ions and organophosphorus pesticides. J. Hazard. Mater..

[133] Kim S.N., Rusling J.F., Papadimitrakopoulos F. (2007). Carbon Nanotubes for Electronic and Electrochemical Detection of Biomolecules. Adv. Mater..

[134] Chu N.-C., Taylor R.N., Chavagnac V.R., Nesbitt R.W., Boella R.M., Milton J.A., German C.R., Bayon G., Burton K. (2002). Hf isotope ratio analysis using multi-collector inductively coupled plasma mass spectrometry: An evaluation of isobaric interference corrections. J. Anal. At. Spectrom..

[135] Willis J.B. (1961). Determination of Lead in Urine by Atomic Absorption Spectroscopy. Nature.

[136] Tao Y., Jiang Y., Huang Y., Liu J., Zhang P., Chen X., Fan Y., Wang L., Xu J. (2021). Carbon dots@metal–organic frameworks as dual-functional fluorescent sensors for Fe^3+^ ions and nitro explosives. CrystEngComm.

[137] Li Y.K., Wei Z.H., Zhang Y., Guo Z.F., Chen D.S., Jia P.Y., Chen P., Xing H.Z. (2019). Dual-Emitting EY@Zr-MOF Composite as Self-Calibrating Luminescent Sensor for Selective Detection of Inorganic Ions and Nitroaromatics. Acs Sustain. Chem. Eng..

[138] Liu M.F., Yu X., Zhong K.X., Chen X.Y., Feng L.J., Yao S. (2022). Dye-encapsulated nanocage-based metal-organic frameworks as luminescent dual-emitting sensors for selective detection of inorganic ions. Appl. Organomet. Chem..

[139] Liu W., Li S.Q., Shao J., Tian J.L. (2020). A dual-emission Acf@bioMOF-1 platform as fluorescence sensor for highly efficient detection of inorganic ions. J. Solid State Chem..

[140] Zhang Z.N., Wei Z.H., Meng F.Y., Su J.L., Chen D.S., Guo Z.F., Xing H.Z. (2020). RhB-Embedded Zirconium-Naphthalene-Based Metal-Organic Framework Composite as a Luminescent Self-Calibrating Platform for the Selective Detection of Inorganic Ions. Chem. Eur. J..

[141] Que E.L., Domaille D.W., Chang C.J. (2008). Metals in Neurobiology: Probing Their Chemistry and Biology with Molecular Imaging. Chem. Rev..

[142] Bora T., Aksoy Ç., Tunay Z., Aydın F. (2015). Determination of trace elements in illicit spice samples by using ICP-MS. Microchem. J..

[143] Cheng W.-L., Sue J.-W., Chen W.-C., Chang J.-L., Zen J.-M. (2009). Activated Nickel Platform for Electrochemical Sensing of Phosphate. Anal. Chem..

[144] Yi K., Zhang X., Zhang L. (2021). Smartphone-based ratiometric fluorescent definable system for phosphate by merged metal−organic frameworks. Sci. Total Environ..

[145] Zhang R., Song B., Yuan J. (2017). Bioanalytical Methods for Hypochlorous Acid Detection: Recent Advances and Challenges. Trends Anal. Chem..

[146] Liu L., Zhu G., Zeng W., Lv B., Yi Y. (2019). Highly sensitive and selective “off-on” fluorescent sensing platform for ClO^−^ in water based on silicon quantum dots coupled with nanosilver. Anal. Bioanal. Chem..

[147] Zhang J., Yang X. (2012). A simple yet effective chromogenic reagent for the rapid estimation of bromate and hypochlorite in drinking water. Analyst.

[148] Xiong J., Xiao Y., Liang J., Sun J., Gao L., Zhou Q., Hong D., Tan K. (2023). Dye-based dual-emission Eu-MOF synthesized by Post-modification for the sensitive ratio fluorescence visualization sensing of ClO. Spectrochim. Acta Part A Mol. Biomol. Spectrosc..

[149] Broza Y.Y., Zhou X., Yuan M., Qu D., Zheng Y., Vishinkin R., Khatib M., Wu W., Haick H. (2019). Disease Detection with Molecular Biomarkers: From Chemistry of Body Fluids to Nature-Inspired Chemical Sensors. Chem. Rev..

[150] Turtoi A., Dumont B., Greffe Y., Blomme A., Mazzucchelli G., Delvenne P., Mutijima E.N., Lifrange E., De Pauw E., Castronovo V. (2011). Novel comprehensive approach for accessible biomarker identification and absolute quantification from precious human tissues. J. Proteome Res..

[151] Zhang L., Wan S., Jiang Y., Wang Y., Fu T., Liu Q., Cao Z., Qiu L., Tan W. (2017). Molecular Elucidation of Disease Biomarkers at the Interface of Chemistry and Biology. J. Am. Chem. Soc..

[152] Yi K., Li H., Zhang X., Zhang L. (2021). Designed Tb(III)-Functionalized MOF-808 as Visible Fluorescent Probes for Monitoring Bilirubin and Identifying Fingerprints. Inorg. Chem..

[153] Song L., Tian F., Liu Z. (2022). Lanthanide doped metal-organic frameworks as a ratiometric fluorescence biosensor for visual and ultrasensitive detection of serotonin. J. Solid State Chem..

[154] Jia L., Chen X., Xu J., Zhang L., Guo S., Bi N., Zhu T. (2020). A smartphone-integrated multicolor fluorescence probe of bacterial spore biomarker: The combination of natural clay material and metal-organic frameworks. J. Hazard. Mater..

[155] Sun N., Yan B. (2018). Fluorescence detection of urinary N-methylformamide for biomonitoring of human occupational exposure to N,N-dimethylformamide by Eu(III) functionalized MOFs. Sens. Actuators B Chem..

[156] Wei Z.H., Chen D.S., Guo Z.F., Jia P.Y., Xing H.Z. (2020). Eosin Y-Embedded Zirconium-Based Metal-Organic Framework as a Dual-Emitting Built-In Self-Calibrating Platform for Pesticide Detection. Inorg. Chem..

[157] Liu L., Chen X.-L., Shang L., Cai M., Cui H.-L., Yang H., Wang J.-J. (2022). Eu3+-postdoped MOFs are used for fluorescence sensing of TNP, TC and pesticides and for anti-counterfeiting ink application. Dye. Pigment..

[158] Cai Y., Zhu H., Zhou W., Qiu Z., Chen C., Qileng A., Li K., Liu Y. (2021). Capsulation of AuNCs with AIE Effect into Metal–Organic Framework for the Marriage of a Fluorescence and Colorimetric Biosensor to Detect Organophosphorus Pesticides. Anal. Chem..

[159] Sun X.Y., Zhang H.J., Sun Q., Gao E.Q. (2021). PB@UiO-67-CDC-(CH_3_)(2) as an Ultrasensitive Ratiometric Fluorescence Sensor: Visible “Turn-On” Effect for Detecting Preservatives and Amino Acids. Cryst. Growth Des..

[160] Xiong T., Zhang Y., Donà L., Gutiérrez M., Möslein A.F., Babal A.S., Amin N., Civalleri B., Tan J.-C. (2021). Tunable Fluorescein-Encapsulated Zeolitic Imidazolate Framework-8 Nanoparticles for Solid-State Lighting. ACS Appl. Nano Mater..

[161] Jia W., Fan R., Zhang J., Zhu K., Gai S., Nai H., Guo H., Wu J., Yang Y. (2022). Home-made multifunctional auxiliary device for in-situ imaging detection and removal of quinclorac residues through MOF decorated gel refills. Chem. Eng. J..

[162] Lu Z., Li M., Chen M., Wang Q., Wu C., Sun M., Su G., Wang X., Wang Y., Zhou X. (2023). Deep learning-assisted smartphone-based portable and visual ratiometric fluorescence device integrated intelligent gel label for agro-food freshness detection. Food Chem..

[163] Pei J., Xuemei H., Jiayu Y., Xinyu S., Tong B., Yuting Z., Li W. (2023). Dual–emission MOF–based ratiometric platform and sensory hydrogel for visible detection of biogenic amines in food spoilage. Sens. Actuators B Chem..

[164] Seemesh Bhaskar (2023). Biosensing Technologies: A Focus Review on Recent Advancements in Surface Plasmon Coupled Emission. Micromachines.

[165] Mondal S., Subramaniam C. (2020). Xenobiotic Contamination of Water by Plastics and Pesticides Revealed through Real-Time, Ultrasensitive, and Reliable Surface-Enhanced Raman Scattering. ACS Sustain. Chem. Eng..

[166] Rai A., Bhaskar S., Ganesh K.M., Ramamurthy S.S. (2022). Hottest Hotspots from the Coldest Cold: Welcome to Nano 4.0. ACS Appl. Nano Mater..

[167] Qin S.-J., Yan B. (2017). A facile indicator box based on Eu^3+^ functionalized MOF hybrid for the determination of 1-naphthol, a biomarker for carbaryl in urine. Sens. Actuators B Chem..

[168] He J.-X., Yuan H.-Q., Zhong Y.-F., Peng X.-X., Xia Y.-F., Liu S.-Y., Fan Q., Yang J.-L., Deng K., Wang X.-Y. (2022). A luminescent Eu3+-functionalized MOF for sensitive and rapid detection of tetracycline antibiotics in swine wastewater and pig kidney. Spectrochim. Acta Part A Mol. Biomol. Spectrosc..

[169] Qin G., Kong Y., Gan T., Ni Y. (2022). Ultrathin 2D Eu^3+^@Zn-MOF Nanosheets: A Functional Nanoplatform for Highly Selective, Sensitive, and Visualized Detection of Organochlorine Pesticides in a Water Environment. Inorg. Chem..

[170] Sun Z., Li Y., Liu J., Zhao Z., Wang F., Wang X. (2022). Lanthanide-Functionalized Metal−Organic Framework as Ratiometric Probe for Selective Detection of 4-NA and Fe^3+^. J. Inorg. Organomet. Polym. Mater..

[171] Yingmin J., Xin X., Wanpeng M., Bing Y. (2022). An Eu3+-functionalized metal–organic framework (Eu@Zn-MOF) for the highly sensitive detection of rotenone in serum. New J. Chem..

[172] Liu C., Wang H., Hu X., Cao Y., Fang G. (2022). Construction of an ECL Detection Platform for Sensitive Detection of Carbaryl Based on an Eu^3+^-Functionalized Metal–Organic Framework Encapsulated with Nanogold. Foods.

[173] Peng X.-X., Bao G.-M., Zhong Y.-F., Zhang L., Zeng K.-B., He J.-X., Xiao W., Xia Y.-F., Fan Q., Yuan H.-Q. (2020). Highly sensitive and rapid detection of thiabendazole residues in oranges based on a luminescent Tb^3+^-functionalized MOF. Food Chem..

[174] Zheng X., Fan R., Song Y., Wang A., Xing K., Du X., Wang P., Yang Y. (2017). A highly sensitive turn-on ratiometric luminescent probe based on postsynthetic modification of Tb^3+^@Cu-MOF for H_2_S detection. J. Mater. Chem. C.

[175] Wu J.-X., Yan B. (2017). A dual-emission probe to detect moisture and water in organics based on green-Tb^3+^ post-coordinated metal−organic frameworks with red-carbon dots. Dalton Trans..

[176] Zhang Y., Li B., Ma H., Zhang L., Zhang W. (2017). An RGH–MOF as a naked eye colorimetric fluorescent sensor for picric acid recognition. J. Mater. Chem. C.

[177] Jiang X.-X., Li P., Zhao M.-Y., Chen R.-C., Wang Z.-G., Xie J.-X., Lv Y.-K. (2022). In situ encapsulation of SQDs by zinc ion-induced ZIF-8 growth strategy for fluorescent and colorimetric dual-signal detection of alkaline phosphatase. Anal. Chim. Acta.

[178] Luo X., Huang G., Bai C., Wang C., Yu Y., Tan Y., Tang C., Kong J., Huang J., Li Z. (2023). A versatile platform for colorimetric, fluorescence and photothermal multi-mode glyphosate sensing by carbon dots anchoring ferrocene metal-organic framework nanosheet. J. Hazard. Mater..

[179] Wong D., Phani A., Homayoonnia S., Park S.S., Kim S., Abuzalat O. (2022). Manipulating Active Sites of 2D Metal–Organic Framework Nanosheets with Fluorescent Materials for Enhanced Colorimetric and Fluorescent Ammonia Sensing. Adv. Mater. Interfaces.

[180] Liu S., Chen Y., Ruan Z., Lin J., Kong W. (2022). Development of label-free fluorescent biosensor for the detection of kanamycin based on aptamer capped metal-organic framework. Environ. Res..

[181] Peng-Chen S., Long Y., Mi Y., Ling-Xiao W., Ming-Tai S., Wei-Jie H., Hua T., Su-Hua W. (2022). Dye-encapsulated metal–organic framework composites for highly sensitive and selective sensing of oxytetracycline based on ratiometric fluorescence. Chem. Pap..

[182] Guo X., Pan Q., Song X., Guo Q., Zhou S., Qiu J., Dong G. (2020). Embedding carbon dots in Eu^3+^-doped metal-organic framework for label-free ratiometric fluorescence detection of Fe^3+^ ions. J. Am. Ceram. Soc..

[183] Peng X.-X., Bao G.-M., Zhong Y.-F., He J.-X., Zeng L., Yuan H.-Q. (2020). Highly selective detection of Cu^2+^ in aqueous media based on Tb^3+^-functionalized metal-organic framework. Spectrochim. Acta Part A Mol. Biomol. Spectrosc..

[184] Ruan B., Yang J., Zhang Y.J., Ma N., Shi D.A., Jiang T., Tsai F.C. (2020). UiO-66 derivate as a fluorescent probe for Fe^3+^ detection. Talanta.

[185] Zhang N., Zhang D., Zhao J., Xia Z. (2019). Fabrication of a dual-emitting dye-encapsulated metal–organic framework as a stable fluorescent sensor for metal ion detection. Dalton Trans..

[186] Yang W., Yang Y., Li H., Lin D., Yang W., Guo D., Pan Q. (2020). Integration of Cd:ZnS QDs into ZIF-8 for enhanced selectivity toward Cu^2+^ detection. Inorg. Chem. Front..

[187] Lin X., Gao G., Zheng L., Chi Y., Chen G. (2013). Encapsulation of Strongly Fluorescent Carbon Quantum Dots in Metal–Organic Frameworks for Enhancing Chemical Sensing. Anal. Chem..

[188] Hou J., Jia P., Yang K., Bu T., Zhao S., Li L., Wang L. (2022). Fluorescence and Colorimetric Dual-Mode Ratiometric Sensor Based on Zr–Tetraphenylporphyrin Tetrasulfonic Acid Hydrate Metal–Organic Frameworks for Visual Detection of Copper Ions. ACS Appl. Mater. Interfaces.

[189] Guo H., Wang X., Wu N., Xu M., Wang M., Zhang L., Yang W. (2021). In-situ Synthesis of Carbon Dots-embedded Europium Metal-Organic Frameworks for Ratiometric Fluorescence Detection of Hg^2+^ in Aqueous Environment. Anal. Chim. Acta.

[190] Hao J.-N., Yan B. (2014). Highly sensitive and selective fluorescent probe for Ag+ based on a Eu^3+^ post-functionalized metal–organic framework in aqueous media. J. Mater. Chem. A.

[191] Wu X., Xi J., Wei X., Yin C. (2023). An ultra-fast UV-electrochemical sensor based on Cu-MOF for highly sensitive and selective detection of ferric ions. Analyst.

[192] Zhang X., Feng L., Ma S., Xia T., Jiao F., Kong Z., Duan X. (2022). A microporous Tb-based MOF for multifunctional detection of the α-CHC, Cu^2+^ and Fe^3+^. J. Solid State Chem..

[193] Guan Y., Zhao X.-L., Li Q.-X., Huang L., Yang J.-M., Yang T., Yang Y.-H., Hu R. (2021). Iodide-enhanced Cu-MOF nanomaterials for the amplified colorimetric detection of Fe^3+^. Anal. Methods.

[194] Tan S.C., Chin S.F., Pang S.C. (2017). Disposable Carbon Dots Modified Screen Printed Carbon Electrode Electrochemical Sensor Strip for Selective Detection of Ferric Ions. J. Sens..

[195] Kim D.-Y., Shinde S., Saratale R., Syed A., Ameen F., Ghodake G. (2017). Spectrophotometric determination of Fe(III) by using casein-functionalized gold nanoparticles. Microchim. Acta.

[196] Chen L., Ma N., Park Y., Jin S., Hwang H., Jiang D., Jung Y.M. (2017). Highly sensitive determination of iron (III) ion based on phenanthroline probe: Surface-enhanced Raman spectroscopy methods. Spectrochim. Acta Part A Mol. Biomol. Spectrosc..

[197] Olgaç N., Karakuş E., Şahin Y., Liv L. (2021). Voltammetric Method for Determining Ferric Ions with Quercetin. Electroanalysis.

[198] Kalhori S., Ahour F., Aurang P. (2023). Determination of trace amount of iron cations using electrochemical methods at N, S doped GQD modified electrode. Sci. Rep..

[199] Wen X.-Z., Yu H., Ma Y.-J. (2019). Separation and indirect ultraviolet detection of ferrous and trivalent iron ions by using ionic liquids in ion chromatography. J. Sep. Sci..

[200] Yap S.H.K., Chan K.K., Zhang G., Tjin S.C., Yong K.-T. (2019). Carbon Dot-functionalized Interferometric Optical Fiber Sensor for Detection of Ferric Ions in Biological Samples. ACS Appl. Mater. Interfaces.

[201] Anas N.A.A., Fen Y.W., Yusof N.A., Omar N.A.S., Daniyal W.M.E.M.M., Ramdzan N.S.M. (2020). Highly sensitive surface plasmon resonance optical detection of ferric ion using CTAB/hydroxylated graphene quantum dots thin film. J. Appl. Phys..

[202] Zheng Y., Wang X., Guan Z., Deng J., Liu X., Li H., Zhao P. (2022). Application of CD and Eu^3+^ Dual Emission MOF Colorimetric Fluorescent Probe Based on Neural Network in Fe3+ Detection. Part. Part. Syst. Charact..

[203] Manoj D., Theyagarajan K., Saravanakumar D., Senthilkumar S., Thenmozhi K. (2017). Aldehyde functionalized ionic liquid on electrochemically reduced graphene oxide as a versatile platform for covalent immobilization of biomolecules and biosensing. Biosens. Bioelectron..

[204] Devaraj M., Deivasigamani R.K., Jeyadevan S. (2012). Enhancement of the electrochemical behavior of CuO nanoleaves on MWCNTs/GC composite film modified electrode for determination of norfloxacin. Colloids Surf. B Biointerfaces.

[205] He W.-y., Wang K.-p., Yang J.-y. (2018). Spectrophotometric methods for determination of vanadium: A review. Toxicol. Environ. Chem..

[206] Li D., Xu X., Li Z., Wang T., Wang C. (2020). Detection methods of ammonia nitrogen in water: A review. Trends Anal. Chem..

[207] Singh P., Singh M.K., Beg Y.R., Nishad G.R. (2018). A review on spectroscopic methods for determination of nitrite and nitrate in environmental samples. Talanta.

[208] Dutta Choudhury S., Badugu R., Lakowicz J.R. (2015). Directing Fluorescence with Plasmonic and Photonic Structures. Acc. Chem. Res..

[209] Gryczynski I., Malicka J., Gryczynski Z., Lakowicz J.R. (2004). Radiative decay engineering 4. Experimental studies of surface plasmon-coupled directional emission. Anal. Biochem..

[210] Chao L., Di X., Xuan D., Qing H. (2022). A review: Research progress of SERS-based sensors for agricultural applications. Trends Food Sci. Technol..

[211] Shintaro P., Tianxi Y., Lili H. (2016). Review of surface enhanced raman spectroscopic (SERS) detection of synthetic chemical pesticides. Trends Anal. Chem..

[212] Sun X., Li H. (2016). A Review: Nanofabrication of Surface-Enhanced Raman Spectroscopy (SERS) Substrates. Curr. Nanosci..

[213] Michalski R., Pecyna-Utylska P., Kernert J. (2021). Determination of ammonium and biogenic amines by ion chromatography. A review. J. Chromatogr. A.

[214] Paull B., Barron L. (2004). Using ion chromatography to monitor haloacetic acids in drinking water: A review of current technologies. J. Chromatogr. A.

[215] Chang C.-C. (2021). Recent Advancements in Aptamer-Based Surface Plasmon Resonance Biosensing Strategies. Biosensors.

[216] Lakowicz J.R., Ray K., Chowdhury M., Szmacinski H., Fu Y., Zhang J., Nowaczyk K. (2008). Plasmon-controlled fluorescence: A new paradigm in fluorescence spectroscopy. Analyst.

[217] Meng L., Yang Z. (2018). Directional surface plasmon-coupled emission of tilted-tip enhanced spectroscopy. Nanophotonics.

[218] Hoang Minh N., Yoon J.S., Kang D.H., Yoo Y.-E., Kim K. (2023). Assembling Vertical Nanogap Arrays with Nanoentities for Highly Sensitive Electrical Biosensing. Langmuir.

